# Schistosome and malaria exposure and urban–rural differences in vaccine responses in Uganda: a causal mediation analysis using data from three linked randomised controlled trials

**DOI:** 10.1016/S2214-109X(24)00340-1

**Published:** 2024-10-16

**Authors:** Agnes Natukunda, Gyaviira Nkurunungi, Ludoviko Zirimenya, Jacent Nassuuna, Christopher Zziwa, Caroline Ninsiima, Josephine Tumusiime, Ruth Nyanzi, Milly Namutebi, Fred Kiwudhu, Govert J van Dam, Paul L A M Corstjens, Robert Kizindo, Ronald Nkangi, Joyce Kabagenyi, Beatrice Nassanga, Stephen Cose, Anne Wajja, Pontiano Kaleebu, Alison M Elliott, Emily L Webb, POPVAC trial team, Mirriam Akello, Florence A Akello, Hellen Akurut, Susan Amongi, Rebecca Amongin, Barbara Apule, Stephen Cose, Emmanuella Driciru, Alison M Elliott, Joyce Kabagenyi, Joel Kabali, Grace Kabami, Prossy N Kabuubi, Ayoub Kakande, Pontiano Kaleebu, Charity Katushabe, John Kayiwa, Samuel Kiwanuka, Fred Kiwudhu, Robert Kizindo, Moses Kizza, Christine Kukundakwe, Alex Mutebe, Esther Nakazibwe, Loyce Namusobya, Milly Namutebi, Christine Nankabirwa, Beatrice Nassanga, Jacent Nassuuna, Agnes Natukunda, Doreen Nayebare, Caroline Ninsiima, Ronald Nkangi, Gyaviira Nkurunungi, Denis Nsubuga, Ruth Nyanzi, Gloria Oduru, Caroline Onen, Joel Serubanja, Moses Sewankambo, Josephine Tumusiime, Pius Tumwesige, Anne Wajja, Bridgious Walusimbi, Emily L Webb, Ludoviko Zirimenya, Christopher Zziwa

**Affiliations:** a Immunomodulation and Vaccines Focus Area, Vaccine Research Theme, Medical Research Council/Uganda Virus Research Institute and London School of Hygiene and Tropical Medicine Uganda Research Unit, Entebbe, Uganda; b Department of Infection Biology, London School of Hygiene and Tropical Medicine, London, UK; c International Statistics and Epidemiology Group, Department of Infectious Disease Epidemiology, London School of Hygiene and Tropical Medicine, London, UK; d Department of Clinical Research, London School of Hygiene and Tropical Medicine, London, UK; e Department of Parasitology, Leiden University Medical Center, Leiden, Netherlands; f Department of Cell and Chemical Biology, Leiden University Medical Center, Leiden, Netherlands; g Department of Immunology and Molecular Biology, School of Biomedical Sciences, College of Health Sciences, Makerere University, Kampala, Uganda; h Department of Global Health and Amsterdam Institute for Global Health and Development, Amsterdam University Medical Centers, Amsterdam, Netherlands

## Abstract

**Background:**

Vaccine immunogenicity and effectiveness vary geographically. Chronic immunomodulating parasitic infections including schistosomes and malaria have been hypothesised to be mediators of geographical variations.

**Methods:**

We compared vaccine-specific immune responses between three Ugandan settings (schistosome-endemic rural, malaria-endemic rural, and urban) and did causal mediation analysis to assess the role of *Schistosoma mansoni* and malaria exposure in observed differences. We used data from the control groups of three linked randomised trials investigating the effects of intensive parasite treatment among schoolchildren. All participants received the BCG vaccine (week 0); yellow fever (YF-17D), oral typhoid (Ty21a), human papillomavirus (HPV; week 4); and HPV booster and tetanus–diphtheria (week 28). Primary outcomes were vaccine responses at week 8 and, for tetanus–diphtheria, week 52. We estimated the total effect (TE) of setting on vaccine responses and natural indirect effect (NIE) mediated through current or previous infection with *S mansoni* or malaria, and baseline vaccine-specific responses.

**Findings:**

We included 239 (43%) participants from the schistosomiasis-endemic setting, 171 (30%) from the malaria-endemic setting, and 151 (27%) from the urban setting. At week 8, vaccine responses were lower in rural settings: schistosomiasis-endemic versus urban settings (TE geometric mean ratio for YF-17D plaque reduction neutralisation at 50% (PRNT_50_) titres 0·58 [95% CI 0·37 to 0·91], for *S* Typhi O-lipopolysaccharide-specific IgG 0·61 [0·40 to 0·93], and for tetanus-specific IgG 0·33 [0·22 to 0·51]); malaria-endemic versus urban settings (YF-17D 0·70 [0·49 to 0·99], *S* Typhi O-lipopolysaccharide-specific IgG 0·29 [0·20 to 0·43], and tetanus-specific IgG 0·53 [–0·35 to 0·80]). However, we found higher BCG-specific IFNγ responses in the malaria-endemic versus urban setting (1·54 [1·20 to 1·98]). The estimated NIEs of setting on vaccine responses mediated through previous and current *S mansoni* and malaria were not statistically significant. For malaria-endemic versus urban settings, baseline vaccine-specific responses contributed to some but not all differences: *S* Typhi O-lipopolysaccharide-specific IgG at week 8 (57.9% mediated [38·6 to 77·2]) and week 52 (70·0% mediated [49·4 to 90·6]) and BCG at week 52 (46.4% mediated [–4·8 to 97·7]).

**Interpretation:**

We found significant variation in vaccine response between urban and rural settings but could not confirm a causal role for schistosome or malaria exposure. Other exposures require consideration.

**Funding:**

UK Medical Research Council.

## Introduction

Infectious diseases remain a major burden in low-income countries (LICs), and outbreaks pose a global threat. Effective vaccines are essential for control and elimination. However, evidence over several decades has shown global and local variability in immunogenicity and efficacy of several vaccines, with impaired response more likely in tropical LICs compared with in high-income countries and in rural compared with in urban settings.

BCG vaccine efficacy and responsiveness differ internationally^1^ and regionally,^2^ with reduced efficacy closer to the equator. There is consistent evidence that oral cholera, rotavirus, and polio vaccine responsiveness is reduced in the low-income endemic settings where they are most needed.^3^, ^4^, ^5^, ^6^ Lower neutralising antibody titres following yellow fever vaccine and faster response waning have been seen in Ugandan than in Swiss recipients.^7^ Within-country differences also exist, with lower responses in rural compared with urban areas for influenza and tetanus vaccines in Gabon, and for hepatitis B vaccine in Mongolia.^8^, ^9^

**Research in context**

**Evidence before this study**

Several vaccines have been found to be less effective in low-income compared with high-income countries and in rural compared with urban low-income tropical settings. We did a literature search to examine the potential contribution of helminths and malaria to geographical differences in vaccine responses. We searched PubMed from inception to Oct 6, 2023, using the following search terms and their synonyms: ((“Helminth infection” OR schisto* OR “malaria infection” OR parasite infection OR malaria exposure OR schisto* exposure OR helminth exposure OR immunomodulating helminth”) AND (“urban-rural differences” OR “rural-urban differences”) AND (“vaccine responses” OR “vaccine immunogenicity” OR “vaccine efficacy”)). The identified primary research studies and narrative review revealed that the contribution of parasitic infections to impaired vaccine-induced immune responses is not well understood. Although some studies suggested that parasitic infections, particularly schistosomiasis, can affect vaccine responsiveness, other studies found no evidence of such an effect. One study reported substantial differences in cytokine responses (interferon gamma and interleukin [IL]-13) to mycobacterial vaccine antigen (purified protein derivative) and cytokine (IL-13) and antibody (IgG) responses to tetanus between rural and urban settings in Uganda. However, current helminth infections did not explain observed differences.

**Added value of this study**

To the best of our knowledge, this study represents the first attempt to explore parasitic infections as mediators for the observed differences in vaccine response between different geographical settings among participants receiving the same vaccine schedule, using causal mediation analysis. Our findings show that vaccine responses vary by geographical setting within the same country. However, we did not show a significant role for schistosome and malaria exposure.

**Implications of all the available evidence**

There are important differences in vaccine responses and efficacy between geographical settings. Although *Schistosoma mansoni* and malaria infections are potent immunomodulators, other pathways might contribute more strongly to these differences. Important factors might include previous exposure to the target pathogens or related infections, or to other environmental and biological exposures. There remains a need for further studies to provide insights into additional pathways through which vaccine response is mediated in different geographical settings.

New vaccines are also affected: responses to candidate tuberculosis,^10^ malaria,^11^ and Ebola virus^12^ vaccines are lower in Africa than elsewhere. Previous exposure to the target pathogen or related organisms might contribute, but analyses implicate broader so-called environmental sensitisation, ^13^ the drivers of which have not been fully explained.

Parasitic infections (including helminths and malaria) are potent immunomodulators^14^, ^15^ and substantial evidence shows that their immunomodulation has non-specific bystander effects on responses to other antigens other than helminths and malaria, including pathogens, vaccines, allergens, and auto-antigens, with major effects on health outcomes.^16^

The Population Differences in Vaccine Responses (POPVAC) programme focused on the effect of two treatable parasitic infections on vaccine response heterogeneity, seeking strategies through which vaccine effectiveness might be optimised for low-income, tropical settings.^17^ POPVAC comprised three trials done in rural schistosomiasis-endemic,^18^ rural malaria-endemic,^19^ and urban communities in Uganda.^20^ The trials shared elements of study design and procedures, allowing comparison of vaccine response outcomes across the settings.

Results from each trial are published separately.^21^, ^22^, ^23^ Here, we compared vaccine responses between settings, to assess urban–rural differences in vaccine responses among Ugandan adolescents. Where differences were seen, we used causal mediation analysis to assess to what degree differences can be explained by differences in current or previous *Schistosoma mansoni* and malaria exposure.

## Methods

### Study design

**Figure 1:**
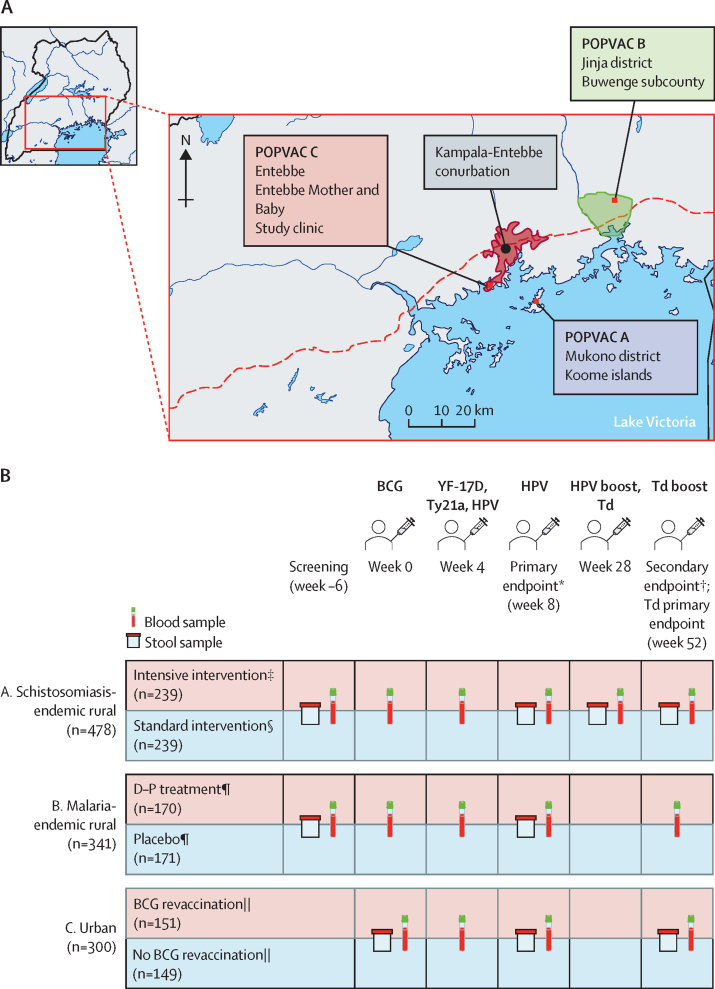
Overview of POPVAC studies (A) POPVAC study sites. (B) Schedule of vaccinations and timepoints of vaccine responses measurements. Borders highlighted in grey denote trial groups being compared in the analysis. Samples were collected before vaccinations or anthelminthic treatment at relevant timepoints. HPV=human papillomavirus. *Primary timepoint following BCG, YF-17D, Ty21a, and HPV vaccination. †Secondary timepoint following BCG, YF-17D, Ty21a, and HPV vaccination. Additionally, an HPV dose given to previously unvaccinated girls aged 14 years or older. ‡Intensive group received three doses of praziquantel (40 mg/kg), each dose 2 weeks apart before week 0, followed by praziquantel at week 8 and then quarterly praziquantel until week 52. §Standard group received one dose of praziquantel at week 8. ¶Participants received 3-day courses of either monthly dihydroartemisinin–piperaquine or placebo twice prior to week 0 and monthly during the entire follow-up period. ||Participants were randomly assigned to either receive BCG or no BCG 4 weeks before the first of the other vaccinations. Created with BioRender.com.

We did a secondary analysis of data from children and adolescents participating in three randomised trials in purposely selected contrasting Ugandan settings (figure 1A). POPVAC A was done in Koome Islands, Mukono district, a schistosomiasis-endemic rural area.^24^ POPVAC B was done in Buwenge county, Jinja district, a malaria-endemic rural area.^25^ POPVAC C was done in Entebbe municipality, Wakiso district, an urban area with lower schistosomiasis and malaria prevalence.

POPVAC A investigated intensive versus standard presumptive praziquantel treatment of *S mansoni* infection on vaccine responses among children aged 9–17 years. Participants in the intensive-treatment group received three praziquantel doses (40 mg/kg) before vaccination, followed by quarterly praziquantel until 12 months. Participants in the standard-treatment group received one praziquantel dose after week-8 sampling. POPVAC B examined the effect of intermittent dihydroartemisinin–piperaquine treatment of malaria versus placebo among school-going children aged 9–17 years. Participants were randomly assigned to monthly dihydroartemisinin–piperaquine or two doses of placebo before vaccination and during the entire follow-up period. POPVAC C studied the effect of BCG re-vaccination on responses to unrelated vaccines among children aged 13–17 years recruited from the Entebbe Mother and Baby Study (EMaBS) birth cohort^26^ and randomly assigned to either receive BCG or no BCG 4 weeks before the first of the other vaccinations.

The study design of the three trials was aligned, with five vaccines administered (figure 1B): live parenteral (BCG and yellow fever [YF-17D] at week 0; live oral (typhoid [Ty21a]); virus-like particle (human papillomavirus [HPV]) at week 4, boosted at week 28; and toxoid (tetanus–diphtheria) at week 28. Full trial design details are described elsewhere.^18^, ^19^, ^20^

### Participants

Participants were included if they were in good health and excluded if they had received study vaccines before enrolment or had any serious illnesses at any time. Detailed eligibility criteria are in the appendix 1 (p 19).

For this analysis comparing responses between geographical settings, we included only participants from the standard group of the schistosomiasis-endemic rural trial (POPVAC A), the placebo group of the malaria-endemic rural trial (POPVAC B), and the BCG re-vaccination group of the urban trial (POPVAC C). We therefore ensured that participants who were compared had received the same schedule of interventions and vaccines. Participants who did not receive vaccines under investigation or had received study vaccines before enrolment were excluded.

### Outcomes, mediators, and other variables

Outcomes were BCG-specific interferon gamma (IFNγ) ELISpot responses, titres of neutralising antibodies against yellow fever virus, *Salmonella enterica* serovar Typhi O-lipopolysaccharide-specific IgG, HPV type 16 and type 18 L1 protein-specific IgG concentration, tetanus toxoid-specific IgG, and diphtheria toxoid-specific IgG. These outcomes were measured at three timepoints (week 0 [baseline], week 8 [primary outcome timepoint], and week 52 [secondary outcome timepoint]), except for tetanus-specific IgG and diphtheria-specific IgG, where measurements were taken at week 28 (baseline) and week 52 (primary outcome timepoint; figure 1B). Details of vaccine-specific response measurement are in the appendix 1 (pp 23–27).

Mediators of interest were current or previous *S mansoni* infection, and current or previous *Plasmodium falciparum* infection*.* We also assessed vaccine-specific baseline responses as potential mediators. The infections were assessed before vaccines were administered. Current *S mansoni* infection was determined as circulating plasma anodic antigen (CAA) at least 30 pg/mL or stool *S mansoni* DNA detected by PCR assay, or both; current malaria (*P falciparum*) infection status by PCR; previous *S mansoni* by ELISA for plasma *Schistosoma* egg antigen-specific IgG; previous malaria by ELISA for plasma IgG against *P falciparum* merozoite surface protein-2 (PfMSP-2) and apical membrane antigen-1 (Pf*AMA-1;* appendix 1 p 28). We examined mediating effects under two scenarios: current and previous *S mansoni* infection and vaccine-specific baseline responses as mediators, comparing vaccine responses between schistosomiasis-endemic rural and urban settings; and current and previous malaria infection and vaccine-specific baseline responses as mediators, comparing malaria-endemic rural and urban settings.

Potential confounders collected at baseline included age and sex, BMI, anthelminthic treatment history, environment (village, or town or city) where the participant lived at birth and aged 5 years.

### Statistical analysis

We created a directed acyclic graph (DAG; appendix 1 p 16) to explicitly illustrate the hypothesised causal pathway between exposure (geographical setting) and outcomes (vaccine responses). To inform the DAG, we did a literature search to identify potential confounders and guide the direction of relationships between variables. The DAG was used to determine our minimal adjustment set (ie, variables to include in regression models). As guided by the DAGitty graphical tool, adjusting for age and sex was sufficient to close all biasing paths and facilitate causal interpretations of regression estimates.^27^

We summarised descriptive statistics (medians [IQR] for continuous variables and frequency and n (%) for categorical variables) of participant characteristics. We used χ^2^ (for categorical variables) and ANOVA tests (for continuous variables) to assess whether characteristics differed significantly between the three study settings. Because vaccine responses had skewed distributions, responses were log_10_ transformed for analysis. To assess whether there were differences in vaccine responses between settings, we used linear regression modelling, adjusted for age and sex. Geometric Mean Ratios (GMR) and 95% CIs are presented.

Where a statistically significant difference was observed between settings, we did causal mediation analyses based on the counterfactual framework^28^ using parametric regression models. This framework uses counterfactual definitions to establish the concepts of natural direct effect (NDE) and natural indirect effects (NIE) via the mediator and builds on the traditional statistical mediation analysis called the product method, also known as the Baron and Kenny method.

The NDE estimates the effect of setting on vaccine responses that does not act through the mediator. The NIE estimates the effect of setting on vaccine responses that acts through the mediator. The total effect is the sum of the direct and the indirect effects and represents the overall effect of setting regardless of whether or not this is mediated by the mediator of interest. An illustration of the decomposition of the total effect into NDE and NIE is presented in the appendix 1 (p 19).

We used the Stata (version 18.0) command “mediate” to estimate GMRs for the NDE, NIE, and total effect (TE), and the proportion mediated (PM) with 95% CIs. Participants with missing vaccine responses and information on mediators were excluded from analyses. For the NDE and NIE estimates to have a causal interpretation, the following assumptions must be met: (1) no uncontrolled exposure-outcome confounding, (2) no uncontrolled mediator-outcome confounding, (3) no uncontrolled exposure-mediator confounding, and (4) no mediator-outcome confounders should be affected by the exposure. Since these assumptions are not directly verifiable from the data, we did a sensitivity analysis to assess the importance of possible violation of no unmeasured confounding assumptions using the mediation E-value statistic.^29^

### Role of the funding source

The funder of the study had no role in study design, data collection, data analysis, data interpretation, or writing of the report.

## Results

Recruitment and follow up for each trial are given in the appendix 1 (p 17). The analysis included 239 (43%) participants from the schistosomiasis-endemic setting, 171 (30%) from the malaria-endemic setting, and 151 (27%) from the urban setting. The median (interquartile range) age of participants in the urban setting was 15 years (IQR 15–16), compared with 11 years (10–13) in the schistosomiasis-endemic setting and 13 years (11–14) in the malaria-endemic setting. 88 (58%) of 151 participants in the urban setting were male and 63 (42%) were female, compared with 136 (57%) of 239 male participants and 103 (43%) female in the schistosomiasis-endemic setting, and 72 (42%) of 171 male and 99 (58%) female in the malaria-endemic setting (table 1). We found significant differences in current and previous *S mansoni* and malaria infection between settings, as expected (table 1).

**Table 1:** Participant characteristics by setting

		***Schistosoma mansoni*-endemic rural (n=239)**	**Malaria-endemic rural (n=171)**	**Urban (n=151)**	**p value**
Median age, years		11 (10–13)	13 (11–14)	15 (15–16)	<0·0001
Sex					
	Female	103 (43%)	99 (58%)	63 (42%)	..
	Male	136 (57%)	72 (42%)	88 (58%)	0·0036
Median BMI		16·8 (15·9–17·9)	17·4 (16·1–18·7)	20·2 (18·6–22·0)	<0·0001
Treatment for worms in the past 12 months		185/211 (88%)	51/82 (62%)	101/150 (67%)	<0·0001
Treatment for malaria in the past 12 months		81/229 (35%)	94/166 (57%)	41 (27%)	<0·0001
Residence of participant at birth					
	Village	215/233 (92%)	143/163 (88%)	0	<0·0001
	Town or city	18/233 (8%)	20/163 (12%)	151 (100%)	..
Residence of participant between birth and age 5 years					
	Village	224/235 (95%)	144/165 (87%)	17/150 (11%)	<0·0001
	Town or city	11/235 (5%)	21/165 (13%)	133/150 (89%)	..
Median baseline antigen-specific responses					
	BCG-specific IFNγ, SFUs/1 million PBMCs	62·5 (35·0–103·3); n=142	85·0 (45·0–155·0); n=126	46·7 (26·7–83·3); n=141	0·0009
	Yellow fever PRNT_50_ titres, proportion above 10	15 (7%); n=219	14 (9%); n=152	0	0·0012
	Yellow fever PRNT_90_ titres, proportion above 10	13 (6%); n=219	8 (5%); n=152	0	0·011
	*S* Typhi O-lipopolysaccharide-specific IgG, EU/mL	115·8 (50·6–282·9); n=219	74·6 (39·7–126·6); n=152	232·3 (93·4–425·9)	<0·0001
	HPV-16-specific IgG, EU/mL*	3·7 (2·5–5·5)	4·6 (2·8–7·3)	3·0 (2·2–5·2)	0·014
	HPV-18-specific IgG, EU/mL*	62·0 (41·4–87·6)	75·6 (41·4–108·0)	60·2 (43·4–91·2)	0·14
	Tetanus toxoid-specific IgG, IU/mL	0·062 (0·032–0·149); n=172	0·075 (0·042–0·173); n=145	0·164 (0·068–0·713); n=146	0·0034
	Diphtheria toxoid-specific IgG, IU/mL	0·044 (0·008–0·198); n=172	0·151 (0·065–0·287); n=145	0·074 (0·018–0·228); n=146	0·016
Current infections					
*Schistosoma mansoni* infection, CAA ≥30 pg/mL					
	Positive	124 (52%); n=238	6 (4%)	26 (17%)	<0·0001
	Negative	114 (48%); n=238	165 (96%)	125 (83%)	..
*S mansoni* infection, PCR					
	Positive	133 (56·1%); n=237	39 (22·9%); n=170	48 (32·4%); n=148	<0·0001
	Negative	104 (43·9%); n=237	131 (77·1%); n=170	100 (67·6%); n=148	..
*S mansoni* infection, CAA or PCR					
	Positive	164 (69%)	43 (25%)	58 (38%)	<0·0001
	Negative	75 (31%)	128 (75%)	93 (62%)	..
Necator americanus, PCR					
	Positive	57 (24%); n=237	17 (10%); n=170	1 (<1%); n=148	<0·0001
	Negative	176 (76%); n=237	153 (90%); n=170	147 (99%); n=148	..
*Strongyloides stercoralis*, PCR					
	Positive	20 (8%); n=237	2 (1%); n=170	0/148	<0·0001
	Negative	217 (92%); n=237	168 (99%); n=170	148/148 (100%)	..
Malaria infection					
	Positive	48 (20%)	99 (58%); n=170	12 (8%)	<0·0001
	Negative	191 (80%)	71 (41%); n=170	139 (92%)	..
Antibody responses representing previous exposure to *S mansoni* and malaria infections					
	Median *Schistosoma* egg antigen-specific IgG, ng/mL	155·5 (58·5–329·5)	36·0 (22·0–57·5)	64·5 (32·0–156·0)	<0·0001
	Median PfMSP-2-specific IgG	234·1 (151·6–378·4)	510·3 (233·2–1494·0)	194·8 (119·1–251·8)	0·0001
	Median PfAMA-1-specific IgG	100·5 (38·1–474·9)	1016·3 (240·1–4194·9)	29·5 (17·6–60·1)	<0·0001

Data are n/N (%), n (%), or median (IQR), unless otherwise indicated. CAA=circulating plasma anodic antigen. HPV=human papillomavirus. PfAMA-1=*Plasmodium falciparum* apical membrane antigen-1. PfMSP-2=*Plasmodium falciparum* merozoite surface protein-2. PRNT_50_=plaque reduction neutralisation at 50%. PRNT_90_=plaque reduction neutralisation at 90%.

* For POPVAC B, these findings exclude girls who had received HPV vaccine before the study. HPV vaccination before the study was an exclusion criterion for all three trials. However, at the time of recruitment for POPVAC B, there had been an HPV vaccination outreach in the study area and to meet the sample size, we opted to recruit some girls who had already received the HPV vaccine.

**Figure 2:**
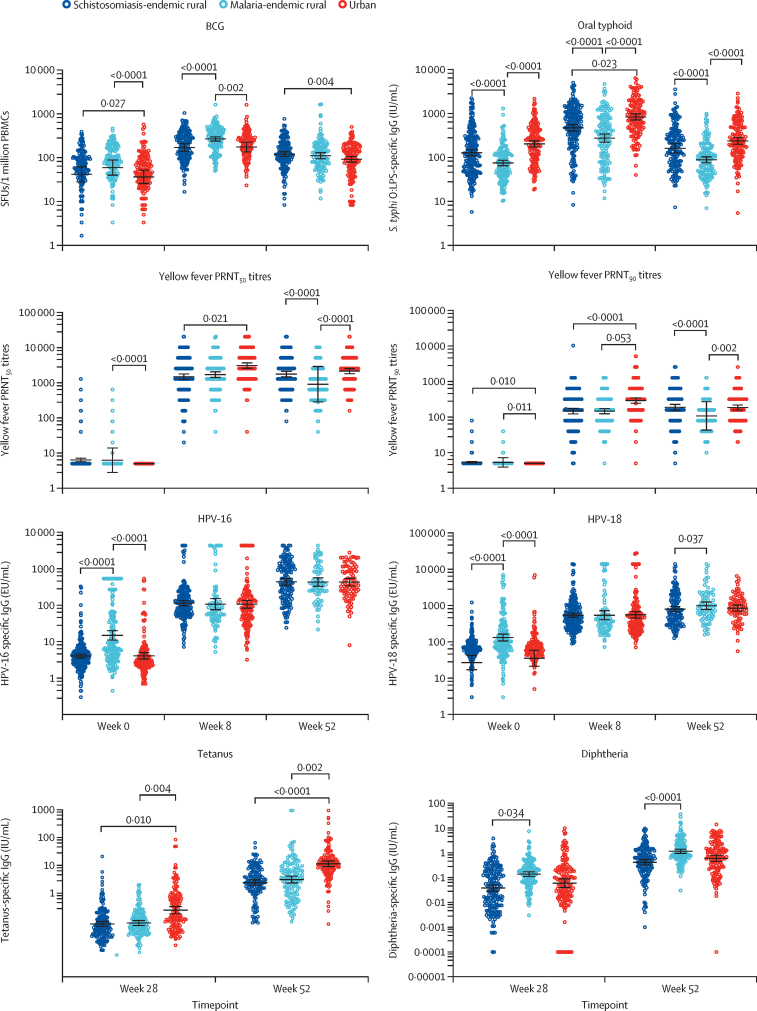
Vaccine responses by geographical setting Plots show individual datapoints, with a horizontal line deonotic the geometric mean and whiskers the 95% CI. SFU=ELISpot assay spot forming unit. PBMC=peripheral blood mononuclear cell. PRNT_50_=plaque reduction neutralisation at 50%. HPV=human papillomavirus.

Before study vaccinations, malaria-endemic rural setting participants had higher baseline BCG-specific IFNɣ and HPV-specific IgG, but lower *S Typhi* O-lipopolysaccharide-specific IgG than urban participants (table 1; figure 2). Participants in rural settings had higher baseline yellow fever neutralising antibody titres, but lower tetanus toxoid-specific IgG, compared with those in rural settings.

Compared with urban participants, post-vaccination responses revealed higher BCG-specific IFNɣ in malaria-endemic rural participants at the primary outcome timepoint and in schistosomiasis-endemic rural participants at the secondary outcome timepoint (figure 2). Yellow fever titres were lower in both rural settings at the primary outcome timepoint and lower in the malaria-endemic setting at the secondary outcome timepoint compared with the urban setting (figure 2). *S* Typhi O-lipopolysaccharide-specific IgG and tetanus toxoid-specific IgG responses were lower in both rural settings at both primary and secondary outcome timepoints compared with the urban setting (figure 2). We found no urban versus rural differences in diphtheria-specific IgG. HPV-16-specific and HPV-18-specific IgG responses were similar between the rural and urban settings, except for slightly higher HPV-18-specific IgG responses in the malaria-endemic rural versus urban setting.

Table 2 shows estimated total, direct, and indirect effects of setting on vaccine responses following study-administered vaccination, comparing the schistosomiasis-endemic rural and urban settings, with *S mansoni* infection as a mediator of indirect effects. Responses for several vaccines were lower in the schistosomiasis-endemic rural than in the urban setting: TE GMR 0·58 (95% CI 0·37–0·91) for YF-17D plaque reduction neutralisation at 50% (PRNT_50_), 0·55 (0·35–0·88) for YF-17D PRNT_90_, 0·61 (0·40–0·93) for *S* Typhi O-lipopolysaccharide-specific IgG 4 weeks after vaccination, and 0·33 (0·22–0·51) for tetanus-specific IgG 24 weeks after vaccination. We found no differences for BCG-specific IFNγ, HPV-16-specific and HPV-18-specific IgG, and diphtheria toxoid-specific IgG at the primary outcome timepoint. However, at the 52-week secondary outcome timepoint, we found higher BCG-specific IFNγ responses in the schistosomiasis-endemic rural compared with the urban setting (1·70 [1·21–2·38]). NIEs of setting on vaccine responses mediated through current *S mansoni* infection were not significantly different from zero for any vaccine response assessed (table 2). Additionally, we found no significant mediating effect of *S mansoni* infection intensity (appendix 1 p 5) and *Schistosoma* egg antigen-specific IgG concentration (appendix 1 p 6).

**Table 2:** Estimates comparing schistosomiasis-endemic rural to urban mediated by *Schistosoma mansoni* infection

	**Total effect, GMR (95% CI)**	**Total effect, p value**	**Natural direct effect, GMR (95% CI)**	**Natural indirect effect, GMR (95% CI)**	**Natural indirect effect, p value**	Proportion mediated (95% CI)*
**Primary outcome timepoint**†						
BCG-specific IFNγ	1·173 (0·846 to 1·625)	0·34	..	..	..	..
Yellow fever PRNT_50_ titres	0·577 (0·366 to 0·907)	0·017	0·522 (0·325 to 0·841)	1·103 (0·954 to 1·276)	0·19	..
Yellow fever PRNT_90_ titres	0·552 (0·347 to 0·877)	0·012	0·501 (0·306 to 0·820)	1·102 (0·957 to 1·269)	0·18	..
*S* Typhi O-lipopolysaccharide-specific IgG	0·614 (0·405 to 0·931)	0·022	0·698 (0·450 to 1·083)	0·880 (0·767 to 1·011)	0·071	26·1% (−10·0 to 62·4)
HPV-16-specific IgG	0·780 (0·495 to 1·230)	0·29	..	..	..	..
HPV-18-specific IgG	0·687 (0·472 to 0·999)	0·050	..	..	..	..
Tetanus toxoid-specific IgG	0·334 (0·218 to 0·511)	<0·001	0·309 (0·200 to 0·476)	1·080 (0·971 to 1·202)	0·16	..
Diphtheria toxoid-specific IgG	1·000 (0·804 to 1·243)	0·99	..	..	..	..
**Secondary outcome timepoint**‡						
BCG-specific IFNγ	1·701 (1·215 to 2·382)	0·002	1·769 (1·255 to 2·492)	0·962 (0·881 to 1·051)	0·39	
Yellow fever PRNT_50_ titres	1·011 (0·608 to 1·681)	0·97	..	..	..	..
Yellow fever PRNT_90_ titres	1·381 (0·804 to 2·373)	0·24	..	..	..	..
*S* Typhi O-lipopolysaccharide-specific IgG	0·733 (0·452 to 1·188)	0·207	..	..	..	..
HPV-16-specific IgG	1·089 (0·598 to 1·986)	0·78	..	..	..	..
HPV-18-specific IgG	0·821 (0·480 to 1·403)	0·47	..	..	..	..

The table shows effects of the urban and rural environment on vaccine responses. Natural indirect effect is the effect of the urban or rural environment mediated though *S mansoni* infection, whereas the natural direct effect is the effect through other pathways. The total effect is the sum of the natural direct and indirect effects. GMR=geometric mean ratio. HPV=human papillomavirus. IFNγ=interferon gamma. PRNT_50_=plaque reduction neutralisation at 50%. PRNT_90_=plaque reduction neutralisation at 90%.

* The proportion mediated was reported only when the total, direct, and indirect effects were in the same direction.

† 8 weeks after BCG vaccination, 4 weeks after 17D, Ty21a, and HPV vaccinations, and 24 weeks after tetanus–diphtheria vaccination.

‡ 52 weeks after BCG vaccination and 48 weeks after 17D, Ty21a, and HPV vaccinations.

Table 3 shows the estimated total, direct, and indirect effects of setting on vaccine responses comparing malaria-endemic rural and urban settings, considering indirect effects mediated through current malaria. At primary outcome timepoints, malaria-endemic rural participants had significantly lower responses for several vaccines compared with those in urban settings: TE GMRs were 0·70 (95% CI 0·49–0·99) for YF-17D PRNT_90_ antibody titres, 0·29 (0·20–0·43) for *S* Typhi O-lipopolysaccharide-specific IgG, and 0·53 (0·35–0·80) for tetanus-specific IgG; however, BCG-specific IFNγ responses were higher (GMR 1·54 [1·20–1·98]). A consistent pattern was observed at the secondary outcome timepoint. NIEs of setting on vaccine responses mediated through current malaria infection were not significantly different from zero for any vaccine response assessed. We also explored previous malaria infection, using PfAMA-1-specific and PfMSP-2-specific antibodies as a potential mediator and found no significant mediating effect (appendix 1 p 7).

**Table 3:** Estimates comparing malaria-endemic rural with urban settings mediated by malaria infection status

	**Total effect, GMR (95% CI)**	**Total effect, p value**	**Natural direct effect, GMR (95% CI)**	**Natural indirect effect, GMR (95% CI)**	**Natural indirect effect, p value**	Proportion mediated (95% CI)*
**Primary outcome timepoint**†						
BCG-specific IFNγ	1·542 (1·199 to 1·984)	0·0008	1·520 (1·144 to 2·020)	1·015 (0·893 to 1·153)	0·82	3·4% (−26·1 to 32·9)
Yellow fever PRNT_50_ titres	0·791 (0·538 to 1·161)	0·23	..	..	..	..
Yellow fever PRNT_90_ titres	0·697 (0·492 to 0·989)	0·043	0·680 (0·446 to 1·035)	1·026 (0·837 to 1·259)	0·804	..
*S* Typhi O-lipopolysaccharide-specific IgG	0·291 (0·196 to 0·432)	<0·0001	0·333 (0·206 to 0·538)	0·874 (0·676 to 1·129)	0·303	10·9% (−10·3 to 32·1)
HPV-16-specific IgG	0·942 (0·573 to 1·550)	0·82	..	..	..	..
HPV-18-specific IgG	0·98 7 (0·641 to 1·520)	0·95	..	..	..	..
Tetanus toxoid-specific IgG	0·529 (0·350 to 0·801)	0·0026	0·470 (0·308 to 0·717)	1·127 (0·911 to 1·395)	0·27	..
Diphtheria toxoid-specific IgG	1·110 (0·891 to 1·383)	0·35	..	..	..	..
**Secondary outcome timepoint**‡						
BCG-specific IFNγ	1·331 (1·012 to 1·750)	0·041	1·200 (0·896 to 1·608)	1·109 (0·937 to 1·312)	0·23	36·1% (−26·1 to 98·3)
Yellow fever PRNT_50_ titres	0·431 (0·284 to 0·655)	<0·0001	0·477 (0·310 to 0·735)	0·904 (0·721 to 1·132)	0·38	12·0% (−14·1 to 38·2)
Yellow fever PRNT_90_ titres	0·574 (0·417 to 0·788)	0·0006	0·547 (0·389 to 0·771)	1·048 (0·876 to 1·253)	0·609	..
*S* Typhi O-lipopolysaccharide-specific IgG	0·413 (0·295 to 0·578)	<0·0001	0·452 (0·306 to 0·667)	0·915 (0·769 to 1·088)	0·31	10·1% (−10·2 to 30·4)
HPV-16-specific IgG	0·630 (0·398 to 0·997)	0·048	0·649 (0·388 to 1·085)	0·971 (0·746 to 1·264)	0·83	6·4% (−50·6 to 63·4)
HPV-18-specific IgG	0·847 (0·566 to 1·270)	0·42	..	..	..	..

The table shows effects of the urban and rural environment on vaccine responses. Natural indirect effect is the effect of the urban or rural environment mediated though malaria infection, whereas the natural direct effect is the effect through other pathways. The total effect is the sum of the natural direct and indirect effects. GMR=geometric mean ratio. HPV= human papillomavirus. IFNγ=interferon gamma. PRNT_50_=plaque reduction neutralisation at 50%. PRNT_90_=plaque reduction neutralisation at 90%.

* The proportion mediated was reported only when the total, direct, and indirect effects were in the same direction.

† 8 weeks after BCG vaccination, 4 weeks after 17D, Ty21a, and HPV vaccinations, and 24 weeks after tetanus–diphtheria vaccination.

‡ 52 weeks after BCG vaccination and 48 weeks after 17D, Ty21a, and HPV vaccinations.

We did further mediation analyses to investigate the role of vaccine-specific baseline responses in the relationship between geographical setting and post-study vaccination responses. Comparing schistosomiasis-endemic rural versus urban settings, we found no significant mediating effect of baseline responses (appendix 1 p 9). Comparing malaria-endemic rural to urban settings, we found a significant role of baseline responses in differences observed for *S* Typhi O-lipopolysaccharide-specific IgG at the primary outcome timepoint (57·9% mediated [95% CI 38·6 to 77·2]), and at the secondary outcome timepoint (70·0% mediated [49·4 to 90·6]). For BCG-specific IFNγ responses, the percentage mediated at the secondary outcome timepoint was 46·4% (–4·8 to 97·7; appendix 1 p 10).

We did sensitivity analyses to assess the extent to which unmeasured or uncontrolled confounding could explain away observed inferences about total and indirect effects. We estimated E-values of 2–5 for significant causal total effects and 1·7–2·9 for significant indirect effects. These values indicate that observed effects of geographical setting on vaccine responses could be explained by an unmeasured or uncontrolled confounder associated with both the setting and vaccine responses by a risk ratio of at least 2–5-times each for the total effects or with the mediator and vaccine responses by a risk ratio of at least 1·7–2·9-times each for indirect effects.

## Discussion

We report substantial differences in vaccine-specific immune responses among Ugandan adolescents receiving the same vaccination schedule in different geographical settings. Causal mediation analysis showed no firm evidence of a mediating role of schistosome or malaria exposure in observed differences, but wide CIs indicate that such a role cannot be conclusively ruled out.

Compared with in the urban setting, children in rural, schistosomiasis-endemic and malaria-endemic settings showed lower YF-17D, Ty21a, and tetanus vaccine responses. By contrast, BCG-specific ELISpot responses were higher in the malaria-endemic setting. HPV and diphtheria showed little difference between settings. These variations highlight the complexity of interactions between geographical setting and vaccine responsiveness, suggesting that elements of the effect might be vaccine-specific.

Although we found no conclusive evidence of mediation by *S mansoni* and malaria, certain points warrant further discussion. Previous studies in animals and humans suggest an impact of *S mansoni* co-infection on some vaccines, particularly BCG;^30^ and that effects may vary, being detrimental for BCG, measles and hepatitis B, and beneficial for polio.^30^ Our schistosomiasis-endemic rural trial^21^ indicated some benefit from treating schistosomiasis for BCG and typhoid vaccine responses; but a detrimental effect of treatment on the priming response to HPV (albeit overridden by the boost). These results accord with a causal role in urban versus schistosomiasis-endemic rural vaccine-response differences, but this finding was not confirmed by the mediation analysis. Contrary to our original hypothesis, BCG-specific ELISpot responses were not reduced in the schistosomiasis-endemic rural compared with in the urban setting; in fact, responses were higher at the secondary outcome timepoint, whereas HPV responses were similar between settings.

For oral typhoid, the response in the schistosomiasis-endemic rural setting was inferior, aligning with an observational analysis within our schistosomiasis-endemic rural trial, where responses to Ty21a were lower in children with higher *S mansoni* intensity. The results from the mediation analysis were consistent with a contribution of *S mansoni,* mediating 26·1% of the lower response in the schistosomiasis-endemic rural setting, albeit with wide CIs.

Although YF-17D and tetanus responses were lower in the schistosomiasis-endemic rural setting, we found no evidence from trial or mediation analyses that this finding is related to *S mansoni*.

In addition to investigating the mediating role of *S mansoni* infection status, we did an exploratory analysis using *S mansoni* intensity based on CAA as a potential mediator and found no significant role of intensity in the vaccine response differences between settings.

Results for comparisons between malaria-endemic rural and urban settings were clear cut, although wide CIs were again observed. Our results from the malaria-endemic rural trial^22^ implied a benefit from malaria treatment for yellow fever PRNT_90_ titres 48 weeks after vaccination, countering the between-setting effect on waning. But we found no evidence of a mediating role for current or past malaria infection in differences between settings.

We found striking differences in baseline vaccine-specific responses between settings. Baseline vaccine-specific responses might be the result of previous vaccination, a consideration in this study for BCG (given at birth) and tetanus–diphtheria, both part of the Uganda National Expanded Programme on Immunisation (EPI). However, previous vaccination would not explain higher baseline BCG-specific or diphtheria-specific responses in rural than in urban settings, as we would expect lower infant vaccination rates in a rural setting, particularly in this POPVAC programme where the urban children were part of a birth cohort provided with EPI vaccines within a research setting, with high coverage.^26^ Yellow fever and typhoid are not part of Uganda's routine immunisations (although deployed in outbreaks). Thus, for all vaccines except tetanus, previous exposure to the target pathogens or cross-reactive organisms is a more likely explanation than previous vaccination for higher baseline responses.

Baseline vaccine-specific responses mediated differences in *S* Typhi O-lipopolysaccharide-specific IgG between malaria-endemic rural and urban settings. We found a contribution of lower baseline tetanus responses to the lower post-vaccination tetanus-specific responses in the malaria-endemic rural versus urban setting. Although not statistically significant, we also found a contribution of higher baseline BCG responses to the higher post-vaccination BCG-specific responses at the primary outcome timepoint and a significant contribution at the secondary outcome timepoint in the malaria-endemic rural setting. Thus, for typhoid, tetanus, and BCG, settings with higher baseline responses generally had higher post-vaccination responses. Overall, mediation effects of baseline responses were much more evident in the malaria-endemic rural versus urban setting than in the *S mansoni*-endemic rural versus urban setting. This finding could be attributed to the stronger baseline differences for BCG and typhoid in the malaria-endemic rural versus urban setting. Contrasting with other vaccines, responses to YF-17D were lower in settings that had higher baseline responses. We found no evidence of a mediating effect of baseline responses, but this observation could be related to broader effects of previous exposure to cross-reactive flaviviruses on the yellow fever response.^31^ Further investigation of characteristics of previous exposure using binding assays for yellow fever and related flaviviruses might clarify this finding.

Although not part of our research question, we explored whether the dominant type of parasitic infection explains differences between the two rural settings. We found that neither active schistosomiasis nor active malaria infection mediated the observed differences (although schistosome-specific serology suggested that overall schistosomiasis exposure might contribute to differences in the response to BCG; appendix 1 p 13).

The strength of this study is that participants from all settings were given the same set of vaccines, and parasitic infection status and vaccine-specific responses were assessed at the same timepoints using the same techniques. Our study has some limitations. First, the sample size was not always sufficient to provide precise estimates for total and indirect effects. We had sufficient power to estimate indirect effects of parasites on vaccine responses between 0·038 and 0·068 log_10_ with a sample size of 320, but insufficient power to detect smaller indirect effects (appendix 1 pp 15, 18). Second, our analysis assumes no unmeasured confounding, which is difficult to achieve in complex epidemiological settings. However, our sensitivity analyses suggested that moderate to strong confounding would be required to explain any causal differences observed. For the schistosomiasis-endemic rural versus urban comparisons, schistosomiasis prevalence in the urban setting was higher than anticipated. Although some level of infection is required in the comparison setting to enable us to assess the infection as a potential mediator, the high schistosomiasis prevalence in the urban setting could have masked its potential mediating role in vaccine response differences between the two settings. In this study, we focused on school-going adolescents because of the high parasite prevalence in this age group, their role as a target group for booster doses of some vaccines and initial doses of others, and the potential utility of combining anti-parasite and vaccine interventions. Our findings might differ if we had focussed on younger age groups—for example, those targeted by EPI vaccines, with different parasite exposure patterns. Finally, our focus was on comparing BCG-specific IFNɣ ELISpot responses, YF-17D neutralising antibody responses, and IgG responses for other vaccines, in line with predefined POPVAC trial outcome measurements; further work will investigate IgG subclasses and other immune parameters to further elucidate possible differences in immune function.

We have shown important differences in vaccine-specific responses between distinct settings even within a small geographical range. We selected settings on the basis of our hypothesis and parasites of interest, anticipating that chronic immunomodulating infections would have a mediating role in urban versus rural differences. Although *S mansoni* and malaria exposure were major distinguishing features of the settings, this analysis could not confirm their causal role. Our study contributes to the growing body of knowledge on vaccine responses in resource-limited regions and underscores the importance of tailoring vaccination strategies to suit the specific populations and their environments. Further research is warranted to validate and expand upon these findings, with a focus on elucidating the complex interplay between environmental and biological factors in explaining geographical differences in vaccine responses.

### POPVAC trial team

### Contributors

### Equitable partnership declaration

### Data sharing

The de-identified individual participant data that underlie the results reported in this Article are stored in a non-publicly available repository (London School of Hygiene and Tropical Medicine [LSHTM] Data Compass), together with a data dictionary accessible via https://doi.org/10.17037/DATA.00003761. Researchers who would like to access the data may submit a request through LSHTM Data Compass, detailing the data requested, the intended use for the data, and evidence of relevant experience and other information to support the request. The request will be reviewed by the Principal Investigator in consultation with the Medical Research Council (MRC) Uganda Virus Research Institute (UVRI) and LSHTM data management committee, with oversight from the UVRI and LSHTM ethics committees. In line with the MRC policy on data sharing, there will have to be a good reason for turning down a request. Patient Information Sheets and consent forms specifically referenced making anonymised data available and this has been approved by the relevant ethics committees. Researchers given access to the data will sign data sharing agreements that will restrict the use to answering pre-specified research questions.

## Declaration of interests

GN and AME report grants from Wellcome Trust. GN reports funding from the EDCTP2 programme supported by the EU. AME and SC report funding from the UK Medical Research Council (MRC) for conduct of the study. AME reports funding from the US National Institutes of Health, Science for Africa Foundation, the Royal Society, and DELTAS Africa, outside the submitted work. AME and AN report support from the UK National Institute of Health and Care Research (NIHR). AME further reports support from the Serum Institute of India, Uganda National Expanded Programme on Immunisation, and Emergent BioSolutions for conduct of the study. BN is currently affiliated with the Jenner Institute, University of Oxford, Oxford, UK. All other authors declare no competing interests.

## Supplementary Materials

Supplementary appendix

Equitable Partnership Declaration
