# Supplementary material for: Schistosome and malaria exposure and urban–rural differences in vaccine responses in Uganda: a causal mediation analysis using data from three linked randomised controlled trials
Source: Lancet Glob Health. 2024 Oct 16;12(11):e1860–70. doi: 10.1016/S2214-109X(24)00340-1 (PMC11483244; doi:10.1016/S2214-109X(24)00340-1)
Supplement: Supplementary appendix [file mmc1.pdf]

THE LANCET
Global Health

Supplementary appendix 1

This appendix formed part of the original submission and has been peer reviewed. We post it as supplied by the authors.

Supplement to: Natukunda A, Nkurunungi G, Zirimenya L, et al. Schistosome and malaria exposure and urban–rural differences in vaccine responses in Uganda: a causal mediation analysis using data from three linked randomised controlled trials. *Lancet Glob Health* 2024; **12:** e1860–70.

SUPPLEMENTARY INFORMATION FOR

Does schistosome or malaria exposure contribute to urban-rural differences in vaccine responses in Uganda? A causal mediation analysis using data from three linked randomised controlled trials.

Agnes Natukunda,* Gyaviira Nkurunungi,* Ludoviko Zirimenya,* Jacent Nassuuna,* Christopher Zziwa, Caroline Ninsiima, Josephine Tumusiime, Ruth Nyanzi, Milly Namutebi, Fred Kiwudhu, Govert J van Dam, Paul LAM Corstjens, Robert Kizindo, Ronald Nkangi, Joyce Kabagenyi, Beatrice Nassanga,[¶] Stephen Cose, Anne Wajja, Pontiano Kaleebu, Alison M Elliott,[#] Emily L Webb,[#] **and the POPVAC trial team†**

Immunomodulation and Vaccines Focus Area, Vaccine Research Theme, Medical Research Council/Uganda Virus Research Institute and London School of Hygiene and Tropical Medicine (MRC/UVRI and LSHTM) Uganda Research Unit, Entebbe, Uganda *(J Nassuuna MSc, L Zirimenya MPH, G Nkurunungi PhD, A Natukunda MSc, C Zziwa BSc, C Ninsiima BSc, J Tumusiime RN, R Nyanzi EN, M Namutebi BSc, F Kiwudhu BBA, R Kizindo, R Nkangi MSc, J Kabagenyi MSc, B Nassanga MSc, S Cose PhD, A Wajja MSc, Prof. P Kaleebu PhD, Prof. AM Elliott MD)*

Department of Infection Biology, London School of Hygiene and Tropical Medicine, London, United Kingdom (*G Nkurunungi PhD*)

International Statistics and Epidemiology Group, Department of Infectious Disease Epidemiology, London School of Hygiene and Tropical Medicine, London, United Kingdom (*A Natukunda MSc, Prof. EL Webb PhD*)

Department of Clinical Research, London School of Hygiene and Tropical Medicine, London, United Kingdom (*L Zirimenya MPH, S Cose PhD, A Wajja MSc, Prof. AM Elliott MD*)

Department of Parasitology, Leiden University Medical Center, Leiden, The Netherlands (*GJ van Dam PhD*)

Department of Cell and Chemical Biology, Leiden University Medical Center, Leiden, The Netherlands (*PLAM Corstjens PhD*)

Department of Immunology and Molecular Biology, School of Biomedical Sciences, College of Health Sciences, Makerere University, Kampala Uganda (*B Nassanga MSc*)

Department of Global Health and Amsterdam Institute for Global Health and Development, Amsterdam University Medical Centers, Amsterdam, Netherlands (*A Wajja MSc*)

*Joint first authors

[#]Joint senior authors

†Trial team members listed at the end of this article

¶Current address: The Jenner Institute, Old Road Campus Research Building, University of Oxford, Oxford, UK

Correspondence to: Dr Gyaviira Nkurunungi, Immunomodulation and Vaccines Focus Area, Vaccine Research Theme, MRC/UVRI and LSHTM Uganda Research Unit, P.O. Box 49, Entebbe, Uganda. **Gyaviira.Nkurunungi@mrcuganda.org**

Table of Contents

Table S1. Estimates comparing schistosomiasis-endemic rural to urban mediated by *S. mansoni* infection intensity measured as CAA concentrations. 5

Table S2. Estimates comparing schistosomiasis-endemic rural to urban mediated by *Schistosoma* egg antigen-specific IgG concentration. 6

Table S3. Estimates comparing malaria-endemic rural to urban mediated by malaria antibodies. 7

Table S4. Estimates comparing schistosomiasis-endemic rural to urban mediated by baseline responses. 9

Table S5. Estimates comparing malaria-endemic rural to urban mediated by baseline responses. 10

Table S6a: Estimates comparing schistosomiasis-endemic rural to malaria endemic rural mediated by *S.mansoni* or malaria infection status 11

Table S6b: Estimates comparing schistosomiasis-endemic rural to malaria endemic rural mediated by *Schistosoma egg antigen* or malaria antibodies 13

Table S7: Indirect effects estimated with power of at least 80% for a sample size of 320 15

Figure S1: Directed acyclic graph of the hypothesised causal relationships between geographical setting and vaccine responses considering parasitic infections as potential mediators. 16

Figure S2: Study profile 17

Figure S3: Power estimates for samples sizes ranging from 230 to 330.............................. 18

Inclusion and exclusion criteria 19

Decomposition of the total effect into natural direct and indirect effects.......................... 19

Plasma detection of *Schistosoma* circulating anodic antigen 20

Stool PCR detection of Schistosoma mansoni, Necator americanus and Strongyloides stercoralis DNA 21

Ex vivo interferon-γ ELISpot assays to quantify BCG-specific responses.............................. 23

Yellow Fever plaque reduction neutralizing reference test (PRNT)...................................... 24

Detection of plasma IgG against *Salmonella* Typhi O-lipopolysaccharide (O:LPS)................ 25

Detection of plasma IgG against Human Papillomavirus type 16 (HPV-16) and HPV-18....... 25

ELISA measurement of anti-diphtheria and anti-tetanus IgG... 26

Detection of *Plasmodium falciparum* DNA by PCR... 27

Detection of IgG against *P. falciparum* merozoite surface protein-2 (MSP-2) and *P. falciparum* apical membrane antigen-1 (AMA-1)... 28

Schistosoma egg antigen (SEA)-specific IgG ELISA.. 28

Supplementary tables

Table S1. Estimates comparing schistosomiasis-endemic rural to urban mediated by *S. mansoni* infection intensity measured as CAA concentrations.

	Total Effect GMR (95% CI)	Total Effect P value	Natural Direct Effect GMR (95% CI)	Natural Indirect Effect GMR (95% CI)	Natural Indirect Effect P value	Proportion mediated % (95% CI)¶
**Primary timepoint***						
BCG-specific IFN-ɣ	1·183(0·857, 1·632)	0·31				
Yellow fever $PRNT_{50}$ titres	0·556(0·347, 0·891)	0·015	0·551(0·344, 0·884)	1.008(0·994, 1·023)	0.25	
Yellow fever $PRNT_{90}$ titres	0·545(0·342, 0·868)	0·011	0·541(0·339, 0·864)	1.007(0·995, 1·019)	0·26	
S. Typhi O: LPS-specific IgG	0·596(0·391, 0·908)	0·016	0·611(0·401, 0·931)	0·975(0·892, 1·065)	0·57	5.0(-12·2, 22·1)
HPV-16-specific IgG	0·830(0·530, 1·299)	0·42				
HPV-18-specific IgG	0·745(0·505, 1·099)	0·14				
Tetanus toxoid-specific IgG	0·306(0·180, 0·520)	0·000	0·307(0·180, 0·524)	0·996(0·983, 1·009)	0·57	0.3(-0.9, 1.5)
Diphtheria toxoid-specific IgG	1·057(0·836, 1·337)	0·64				
**Secondary timepoint#**						
BCG-specific IFN-ɣ	1·836(1·225, 2·752)	0·003	1·847(1·255, 2·719)	0·994(0·959, 1·030)	0·75	
Yellow fever $PRNT_{50}$ titres	0·985(0·586, 1·654)	0·95				
Yellow fever $PRNT_{90}$ titres	1·372(0·792, 2·375)	0·26				
S. Typhi O: LPS-specific IgG	0·790(0·469, 1·1330)	0·38				
HPV-16-specific IgG	1·158(0·620, 2·972)	0·44				
HPV-18-specific IgG	0·919(0·524, 1·611)	0·77				

The table shows effects of the urban-rural environment on vaccine responses. Natural indirect effect is the effect of the urban-rural environment mediated through *S. mansoni* infection intensity whereas the natural direct effect is the effect through other pathways. The total effect is the sum of the natural direct and indirect effects.

GMR: Geometric mean ratio, 95% CI: 95% confidence interval

*8 weeks post-BCG, 4 weeks post- YF-17D, Ty21a and HPV and 24 weeks post-Td

52 weeks post-BCG, 48 weeks post- YF-17D, Ty21a and HPV

¶The proportion mediated was reported only when the total, direct and indirect effects were in the same direction

Table S2. Estimates comparing schistosomiasis-endemic rural to urban mediated by *Schistosoma* egg antigen-specific IgG concentration.

	**Total Effect GMR (95% CI)**	**Total Effect P value**	**Natural Direct Effect GMR (95% CI)**	**Natural Indirect Effect GMR (95% CI)**	**Natural Indirect Effect P value**	**Proportion mediated % (95% CI)¶**
**Primary timepoint***						
BCG-specific IFN-ɣ	1·176(0·853, 1·621)	0·32				
Yellow fever $PRNT_{50}$ titres	0·574(0·365, 0·903)	0·016	0·577(0·368, 0·903)	0·996(0·916, 1·083)	0·93	0·7(-14·4, 15·8)
Yellow fever $PRNT_{90}$ titres	0·551(0·347, 0·873)	0·011	0·558(0·351, 0·887)	0·987(0·912, 1·067)	0·74	2·3(-10·9, 15·4)
S. Typhi O: LPS-specific IgG	0·611(0·401, 0·932)	0·022	0·618(0·408, 0·936)	0·990(0·914, 1·073)	0·809	2·0(-14·1, 18·1)
HPV-16-specific IgG	0·819(0·524, 1·278)	0·38				
HPV-18-specific IgG	0·714(0·493, 1·034)	0·075				
Tetanus toxoid-specific IgG	0·345(0·224, 0·532)	0·000	0·356(0·231, 0·546)	0·971(0·916, 1·030)	0·34	2·7(-2·8, 8·2)
Diphtheria toxoid-specific IgG	1·004(0·806, 1·251)	0·97				
**Secondary timepoint#**						
BCG-specific IFN-ɣ	1·704(1·214, 2·392)	0·002	1·775(1·269, 2·483)	0·960(0·909, 1·014)	0·15	
Yellow fever $PRNT_{50}$ titres	0·989(0·594, 1·645)	0·97				
Yellow fever $PRNT_{90}$ titres	1·352(0·784, 2·331)	0·28				
S. Typhi O: LPS-specific IgG	0·736(0·453, 1·194)	0·214				
HPV-16-specific IgG	1·135(0·625, 2·059)	0·68				
HPV-18-specific IgG	0·850(0·500, 1·445)	0·55				

The table shows effects of the urban-rural environment on vaccine responses. Natural indirect effect is the effect of the urban-rural environment mediated through prior exposure to *S. mansoni* infection whereas the natural direct effect is the effect through other pathways. The total effect is the sum of the natural direct and indirect effects.

GMR: Geometric mean ratio, 95% CI: 95% confidence interval

*8 weeks post-BCG, 4 weeks post- YF-17D, Ty21a and HPV and 24 weeks post-Td

[#] 52 weeks post-BCG, 48 weeks post- YF-17D, Ty21a and HPV

[¶]The proportion mediated was reported only when the total, direct and indirect effects were in the same direction

Table S3. Estimates comparing malaria-endemic rural to urban mediated by malaria antibodies.

	Total Effect GMR (95% CI)	Total Effect P value	Natural Direct Effect GMR (95% CI)	Natural Indirect Effect GMR (95% CI)	Natural Indirect Effect P value	Proportion mediated % (95% CI)¶
**Primary time point***						
**BCG-specific IFN-ɣ**						
PfMSP2 IgG titre	1·525(1·175, 1·978)	0·002	1·479(1·099, 1·991)	1·031(0·928, 1·144)	0·57	7·1(-18·8, 33·1)
PfAMA1 IgG titre	1·542(1·188, 2·002)	0·001	1·464(1·024, 2·093)	1·053(0·851, 1·303)	0·63	12·0(-38·6, 62·6)
**Yellow fever $PRNT_{50}$ titres**						
PfMSP2 IgG titre	0·787(0·532, 1·166)	0·23				
PfAMA1 IgG titre	0·809(0·549, 1·193)	0·28				
**Yellow fever $PRNT_{90}$ titres**						
PfMSP2 IgG titre	0·705(0·489, 1·016)	0·061				
PfAMA1 IgG titre	0·724(0·505, 1·037)	0·078				
***S.* Typhi O: LPS-specific IgG**						
PfMSP2 IgG titre	0·273(0·183, 0·407)	<0·0001	0·263(0·158, 0·435)	1·039(0·787, 1·372)	0·79	
PfAMA1 IgG titre	0·274(0·183, 0·410)	<0·0001	0·336(0·167, 0·678)	0·814(0·495, 1·340)	0·42	15·9(-23·9, 55·6)
**HPV-16-specific IgG**						
PfMSP2 IgG titre	0·976(0·592, 1·609)	0·93				
PfAMA1 IgG titre	1·006(0·606, 1·668)	0·98				
**HPV-18-specific IgG**						
PfMSP2 IgG titre	1·019(0·660, 1·573)	0·93				
PfAMA1 IgG titre	1·004(0·646, 1·559)	0·99				
**Tetanus toxoid-specific IgG**						
PfMSP2 IgG titre	0·509(0·333, 0·779)	0·002	0·500(0·316, 0·792)	1·018(0·824, 1·257)	0·87	
PfAMA1 IgG titre	0·500(0·327, 0·765)	0·001	0·457(0·257, 0·811)	1·095(0·716, 1·675)	0·68	
**Diphtheria toxoid-specific IgG**						
PfMSP2 IgG titre	1·095(0·883, 1·357)	0·410				
PfAMA1 IgG titre	1·084(0·874, 1·344)	0·46				
**Secondary timepoint#**						
**BCG-specific IFN-ɣ**						
PfMSP2 IgG titre	1·333(1·013, 1·754)	0·040	1·532(1·122, 2·093)	0·870(0·719, 1·053)	0·15	
PfAMA1 IgG titre	1·342(1·019, 1·769)	0·036	1·422(0·965, 2·096)	0·944(0·708, 1·259)	0·69	

**Yellow fever PRNT$_{50}$ titres**						
PfMSP2 IgG titre	0·403(0·265, 0·613)	<0·0001	0·378(0·231, 0·617)	1·066(0·869, 1·307)	0·54	
PfAMA1 IgG titre	0·409(0·268, 0·625)	<0·0001	0·362(0·204, 0·640)	1·132(0·774, 1·655)	0·52	
**Yellow fever PRNT$_{90}$ titres**						
PfMSP2 IgG titre	0·560(0·405, 0·774)	<0·0001	0·570(0·383, 0·848)	0·982(0·821, 1·175)	0·84	
PfAMA1 IgG titre	0·556(0·403, 0·769)	<0·0001	0·440(0·275, 0·703)	1·266(0·925, 1·732)	0·14	
***S.* Typhi O: LPS-specific IgG**						
PfMSP2 IgG titre	0·409(0·289, 0·577)	<0·0001	0·392(0·261, 0·588)	1·043(0·863, 1·260)	0·66	
PfAMA1 IgG titre	0·408(0·288, 0·576)	<0·0001	0·461(0·282, 0·754)	0·885(0·646, 1·211)	0·44	13·7(-22·3, 49·6)
**HPV-16-specific IgG**						
PfMSP2 IgG titre	0·618(0·387, 0·987)	0·044				
PfAMA1 IgG titre	0·628(0·400, 0·985)	0·043				
**HPV-18-specific IgG**						
PfMSP2 IgG titre	0·855(0·567, 1·288)	0·45				
PfAMA1 IgG titre	0·858(0·574, 1·283)	0·46				

The table shows effects of the urban-rural environment on vaccine responses. Natural indirect effect is the effect of the urban-rural environment mediated through malaria antibodies whereas the natural direct effect is the effect through other pathways. The total effect is the sum of the natural direct and indirect effects.

GMR: Geometric mean ratio, CI: 95% confidence interval

*8 weeks post-BCG, 4 weeks post- YF-17D, Ty21a and HPV and 24 weeks post-Td

#52 weeks post-BCG, 48 weeks post- YF-17D, Ty21a and HPV

¶The proportion mediated was reported only when the total, direct and indirect effects were in the same direction

Table S4. Estimates comparing schistosomiasis-endemic rural to urban mediated by baseline responses.

	Total Effect GMR (95% CI)	Total Effect P value	Natural Direct Effect GMR (95% CI)	Natural Indirect Effect GMR (95% CI)	Natural Indirect Effect P value	Proportion mediated % (95% CI)[¶]
**Primary timepoint***						
BCG-specific IFN-ɣ	1·162(0·802, 1·684)	0·43				
Yellow fever $PRNT_{50}$ titres	0·557(0·354, 0·879)	0·012	0·557(0·353, 0·879)	1·001(0·976, 1·026)	0·96	
Yellow fever $PRNT_{90}$ titres	0·538(0·338, 0·858)	0·009	0·539(0·338, 0·858)	0·999(0·991, 1·007)	0·87	0·1(-1·2, 1·4)
S. Typhi O: LPS-specific IgG	0·585(0·382, 0·894)	0·013	0·668(0·475, 0·941)	0·875(0·684, 1·119)	0·29	24·9(-12·7, 62·6)
Tetanus toxoid-specific IgG	0·328(0·213, 0·506)	0·000	0·470(0·252, 0·876)	0·698(0·419, 1·161)	0·17	32·3(-13·4, 78·0)
Diphtheria toxoid-specific IgG	0·983(0·786, 1·229)	0·88				
HPV-16-specific IgG	0·846(0·5403, 1·326)	0·47				
HPV-18-specific IgG	0·706(0·469, 1·061)	0·094				
**Secondary time point**[#]						
BCG-specific IFN-ɣ	1·588(1·087, 2·318)	0·017	1·388(0·958, 2·012)	1·144(0·931, 1·405)	0·201	29·0(-14·5, 72·6)
Yellow fever $PRNT_{50}$ titres	0·995(0·592, 1·670)	0·98				
Yellow fever $PRNT_{90}$ titres	1·369(0·790, 2·372)	0·26				
S. Typhi O: LPS-specific IgG	0·753(0·462, 1·228)	0·26				
HPV-16-specific IgG	1·117(0·614, 2·033)	0·72				
HPV-18-specific IgG	0·861(0·502, 1·477)	0·59				

The table shows effects of the urban-rural environment on vaccine responses. Natural indirect effect is the effect of the urban-rural environment mediated through baseline responses whereas the natural direct effect is the effect through other pathways. The total effect is the sum of the natural direct and indirect effects.

GMR: Geometric mean ratio, CI: 95% confidence interval

*8 weeks post-BCG, 4 weeks post- YF-17D, Ty21a and HPV and 24 weeks post-Td

[#]52 weeks post-BCG, 48 weeks post- YF-17D, Ty21a and HPV

[¶]The proportion mediated was reported only when the total, direct and indirect effects were in the same direction

Table S5. Estimates comparing malaria-endemic rural to urban mediated by baseline responses.

	Total Effect GMR (95% CI)	Total Effect P value	Natural Direct Effect GMR (95% CI)	Natural Indirect Effect GMR (95% CI)	Natural Indirect Effect P value	Proportion mediated % (95% CI)[¶]
**Primary timepoint***						
BCG-specific IFN-ɣ	1·550(1·192, 2·016)	0·001	1·448(1·104, 1·898)	1·071(0·988, 1·160)	0·095	15·6(-4·5, 35·8)
Yellow fever $PRNT_{50}$ titres	0·813(0·547, 1·208)	0·306				
Yellow fever $PRNT_{90}$ titres	0·716(0·499, 1·027)	0·070				
S. Typhi O: LPS-specific IgG	0·271(0·183, 0·401)	<0·0001	0·577(0·421, 0·789)	0·469(0·340, 0·647)	<0·0001	57·9(38·6, 77·2)
HPV-16-specific IgG	0·955(0·575, 1·586)	0·86				
HPV-18-specific IgG	0·994(0·642, 1·539)	0·98				
Tetanus toxoid-specific IgG	0·482(0·320, 0·728)	0·001	0·937(0·452, 1·939)	0·515(0·260, 1·019)	0·057	
Diphtheria toxoid-specific IgG	1·110(0·888, 1·387)	0·36				
**Secondary timepoint[#]**						
BCG-specific IFN-ɣ	1·408(1·065, 1·861)	0·016	1·201(0·896, 1·609)	1·172(1·020, 1·347)	0·025	46·4(-4·8, 97·7)
Yellow fever $PRNT_{50}$ titres	0·433(0·281, 0·668)	<0·0001	0·448(0·287, 0·701)	0·966(0·866, 1·078)	0·53	4·1(-9·1, 17·4)
Yellow fever $PRNT_{90}$ titres	0·561(0·405, 0·776)	<0·0001	0·591(0·421, 0·830)	0·948(0·885, 1·016)	0·13	9·1(-4·3, 22·7)
S. Typhi O: LPS-specific IgG	0·415(0·294, 0·585)	<0·0001	0·768(0·611, 0·965)	0·540(0·414, 0·704)	<0·0001	70·0(49·4, 90·6)
HPV-16-specific IgG	0·627(0·389, 1·011)	0·055				
HPV-18-specific IgG	0·822(0·544, 1·243)	0·35				

The table shows effects of the urban-rural environment on vaccine responses. Natural indirect effect is the effect of the urban-rural environment mediated through baseline responses whereas the natural direct effect is the effect through other pathways. The total effect is the sum of the natural direct and indirect effects.

GMR: Geometric mean ratio, CI: 95% confidence interval

*8 weeks post-BCG, 4 weeks post- YF-17D, Ty21a and HPV and 24 weeks post-Td

[#]52 weeks post-BCG, 48 weeks post- YF-17D, Ty21a and HPV

[¶]The proportion mediated was reported only when the total, direct and indirect effects were in the same direction

Table S6a: Estimates comparing schistosomiasis-endemic rural to malaria endemic rural mediated by *S.mansoni* or malaria infection status

	**Total Effect GMR (95% CI)**	**Total Effect P value**	**Natural Direct Effect GMR (95% CI)**	**Natural Indirect Effect GMR (95% CI)**	**Natural Indirect Effect P value**	**Proportion mediated % (95% CI)¶**
**Primary timepoint***						
BCG-specific IFN-ɣ						
S. mansoni as the mediator (A vs B)	0·689(0·580, 0·818)	<0·0001	0·701(0·558, 0·879)	0·983(0·866, 1·116)	0·79	4·6(-29·9, 38·9)
Malaria as the mediator (B vs A)	1·451(1·220, 1·726)	<0·0001	1·435(1·184, 1·741)	1·011(0·930, 1·099)	0·79	3·0(-19·5, 25·4)
Yellow fever $PRNT_{50}$ titres						
S. mansoni (A vs B)	0·961(0·736, 1·253)	0·77				
Yellow fever $PRNT_{90}$ titres						
S. mansoni (A vs B)	1·051(0·814, 1·356)	0·70				
S. Typhi O:LPS-specific IgG						
S. mansoni as the mediator (A vs B)	1·671(1·253, 2·229)	<0·0001	1·997(1·434, 2·781)	0·837(0·704, 0·994)	0·04	
Malaria as the mediator (B vs A)	0·596(0·447, 0·795)	<0·0001	0·659(0·467, 0·932)	0·903(0·756, 1·080)	0·27	19·6(-17·1, 56·2)
Tetanus toxoid-specific IgG						
S. mansoni (A vs B)	0·829(0·636, 1·080)	0·16				
Diphtheria toxoid-specific IgG						
S. mansoni as the mediator (A vs B)	0·686(0·587, 0·800)	<0·0001	0·679(0·579, 0·798)	1·009(0·935, 1·088)	0·82	
Malaria as the mediator (B vs A)	1·459(1·249, 1·703)	<0·0001	1·513(1·252, 1·827)	0·965(0·883, 1·054)	0·43	
HPV-16-specific IgG						
S. mansoni (A vs B)	0·825(0·581, 1·172)	0·28				
HPV-18-specific IgG						
S. mansoni (A vs B)	0·839(0·625, 1·127)	0·25				
**Secondary timepoint#**						
BCG-specific IFN-ɣ						

S. mansoni (A vs B)	1·178(0·959, 1·448)	0·12				
Yellow fever PRNT$_{50}$ titres						
S. mansoni as the mediator (A vs B)	2·281(1·720, 3·024)	<0·0001	2·609(1·906, 3·572)	0·874(0·739, 1·034)	0·12	
Malaria as the mediator (B vs A)	0·438(0·330, 0·582)	<0·0001	0·471(0·339, 0·654)	0·931(0·803, 1·079)	0·34	8·7(-9·7, 27·0)
Yellow fever PRNT$_{90}$ titres						
S. mansoni as the mediator (A vs B)	2·080(1·591, 2·720)	<0·0001	2·232(1·669, 2·983)	0·932(0·786, 1·106)	0·42	
Malaria as the mediator (B vs A)	0·481(0·368, 0·629)	<0·0001	0·471(0·350, 0·633)	1·021(0·909, 1·147)	0·72	
S. Typhi O:LPS-specific IgG						
S. mansoni as the mediator (A vs B)	2·050(1·593, 2·638)	<0·0001	2·244(1·663, 3·030)	0·913(0·767, 1·088)	0·31	
Malaria as the mediator (B vs A)	0·487(0·378, 0·626)	<0·0001	0·518(0·390, 0·687)	0·941(0·834, 1·062)	0·32	8·5(-8·7, 25·7)
HPV-16-specific IgG						
S. mansoni (A vs B)	0·931(0·670, 1·294)	0·67				
HPV-18-specific IgG						
S. mansoni as the mediator (A vs B)	0·735(0·547, 0·988)	0·04	0·695(0·486, 0·993)	1·057(0·886, 1·261)	0·54	
Malaria as the mediator (B vs A)	1·361(1·013, 1·828)	0·04	1·375(0·976, 1·937)	0·989(0·837, 1·169)	0·90	

The table shows effects of the schistosomiasis endemic rural versus malaria endemic rural environments on vaccine responses. Natural indirect effect is the effect of the environment mediated through schistosomiasis or malaria whereas the natural direct effect is the effect through other pathways. The total effect is the sum of the natural direct and indirect effects.
GMR: Geometric mean ratio, CI: 95% confidence interval
*8 weeks post-BCG, 4 weeks post- YF-17D, Ty21a and HPV and 24 weeks post-Td
#52 weeks post-BCG, 48 weeks post- YF-17D, Ty21a and HPV
¶The proportion mediated was reported only when the total, direct and indirect effects were in the same direction

Table S6b: Estimates comparing schistosomiasis-endemic rural to malaria endemic rural mediated by *Schistosoma egg antigen* or malaria antibodies

	**Total Effect GMR (95% CI)**	**Total Effect P value**	**Natural Direct Effect GMR (95% CI)**	**Natural Indirect Effect GMR (95% CI)**	**Natural Indirect Effect P value**	**Proportion mediated % (95% CI)¶**
**Primary timepoint***						
BCG-specific IFN-ɣ						
SEA as the mediator (A vs B)	0·690(0·582, 0·819)	<0·0001	0·785(0·645, 0·956)	0·879(0·809, 0·955)	0·002	34·7(6·1, 63·2)
PfMSP2 as the mediator (B vs A)	1·453(1·179, 1·789)	<0·0001	1·441(1·163, 1·785)	1·008(0·998, 1·018)	0·10	2·2(-1·3, 5·7)
PfAMA1 as the mediator (B vs A)	1·469(1·232, 1·749)	<0·0001	1·431(1·189, 1·722)	1·026(0·972, 1·084)	0·35	6·8(-7·8, 21·4)
Yellow fever $PRNT_{50}$ titres						
(A vs B)	0·961(0·736, 1·253)	0·77				
Yellow fever $PRNT_{90}$ titres						
(A vs B)	1·051(0·814, 1·356)	0·70				
S. Typhi O:LPS-specific IgG						
SEA as the mediator (A vs B)	1·649(1·239, 2·193)	0·001	1·682(1·236, 2·289)	0·980(0·849, 1·132)	0·79	
PfMSP2 as the mediator (B vs A)	0·581(0·432, 0·780)	<0·0001	0·568(0·420, 0·768)	1·022(0·994, 1·051)	0·13	
PfAMA1 as the mediator (B vs A)	0·584(0·436, 0·783)	<0·0001	0·648(0·478, 0·878)	0·902(0·802, 1·015)	0·09	19·1(4·2, 42·4)
Tetanus toxoid-specific IgG						
(A vs B)	0·829(0·636, 1·080)	0·16				
Diphtheria toxoid-specific IgG						
SEA as the mediator (A vs B)	0·683(0·585, 0·797)	<0·0001	0·713(0·608, 0·835)	0·958(0·905, 1·015)	0·14	11·1(-3·9, 26·2)
PfMSP2 as the mediator (B vs A)	1·432(1·230, 1·667)	<0·0001	1·435(1·230, 1·673)	0·998(0·987, 1·009)	0·75	
PfAMA1 as the mediator (B vs A)	1·435(1·234, 1·670)	<0·0001	1·395(1·193, 1·632)	1·028(0·976, 1·083)	0·29	7·8(-6·7, 22·3)
HPV-16-specific IgG						
(A vs B)	0·825(0·581, 1·172)	0·28				
HPV-18-specific IgG						
(A vs B)	0·839(0·625, 1·127)	0·25				
**Secondary timepoint#**						
BCG-specific IFN-ɣ						
(A vs B)	1·178(0·959, 1·448)	0·12				
Yellow fever $PRNT_{50}$ titres						
SEA as the mediator (A vs B)	2·232(1·682, 2·962)	<0·0001	2·639(1·951, 3·570)	0·846(0·744, 0·962)	0·01	

PfMSP2 as the mediator (B vs A)	0·426(0·321, 0·566)	<0·0001	0·429(0·321, 0·572)	0·994(0·980, 1·007)	0·37	0·7(-0·9, 2·4)
PfAMA1 as the mediator (B vs A)	0·425(0·320, 0·563)	<0·0001	0·411(0·306, 0·552)	1·033(0·952, 1·120)	0·44	
Yellow fever $PRNT_{90}$ titres						
SEA as the mediator (A vs B)	2·066(1·579, 2·703)	<0·0001	2·245(1·679, 3·001)	0·920(0·801, 1·058)	0·24	
PfMSP2 as the mediator (B vs A)	0·473(0·361, 0·620)	<0·0001	0·482(0·368, 0·633)	0·981(0·959, 1·003)	0·09	2·6(-0·6, 5·7)
PfAMA1 as the mediator (B vs A)	0·473(0·361, 0·619)	<0·0001	0·449(0·339, 0·594)	1·054(0·982, 1·131)	0·15	
S. Typhi O:LPS-specific IgG						
SEA as the mediator (A vs B)	2·028(1·574, 2·614)	<0·0001	1·977(1·492, 2·620)	1·026(0·899, 1·169)	0·70	
PfMSP2 as the mediator (B vs A)	0·488(0·375, 0·634)	<0·0001	0·485(0·375, 0·629)	1·005(0·969, 1·041)	0·80	
PfAMA1 as the mediator (B vs A)	0·485(0·376, 0·626)	<0·0001	0·493(0·377, 0·646)	0·983(0·917, 1·054)	0·64	2·3(-7·4, 12·0)
HPV-16-specific IgG						
(A vs B)	0·931(0·670, 1·294)	0·67				
HPV-18-specific IgG						
SEA as the mediator (A vs B)	0·731(0·544, 0·982)	0·04	0·678(0·487, 0·944)	1·078(0·950, 1·222)	0·25	
PfMSP2 as the mediator (B vs A)	1·344(0·994, 1·816)	0·05	1·319(0·968, 1·796)	1·019(0·916, 1·134)	0·73	6·3(-29·7, 42·3)
PfAMA1 as the mediator (B vs A)	1·359(1·007, 1·834)	0·05	1·384(1·013, 1·891)	0·982(0·904, 1·067)	0·67	

The table shows effects of the schistosomiasis endemic rural versus malaria endemic rural environments on vaccine responses. Natural indirect effect is the effect of the environment mediated through Schistosoma egg antigen (SEA) or malaria antibodies (MSP2 and PfAMA1) whereas the natural direct effect is the effect through other pathways. The total effect is the sum of the natural direct and indirect effects.

GMR: Geometric mean ratio, CI: 95% confidence interval

*8 weeks post-BCG, 4 weeks post- YF-17D, Ty21a and HPV and 24 weeks post-Td

[#]52 weeks post-BCG, 48 weeks post- YF-17D, Ty21a and HPV

[¶]The proportion mediated was reported only when the total, direct and indirect effects were in the same direction

Table S7: Indirect effects estimated with power of at least 80% for a sample size of 320

	Indirect effect (log_{10}) mean difference
BCG-specific IFN-ɣ	0.042
Yellow fever $PRNT_{50}$ titres	0.057
Yellow fever $PRNT_{90}$ titres	0.056
S. Typhi O:LPS-specific IgG	0.062
HPV-16-specific IgG	0.068
HPV-18-specific IgG	0.060
Tetanus toxoid-specific IgG	0.059
Diphtheria toxoid-specific IgG	0.038

Supplementary figures

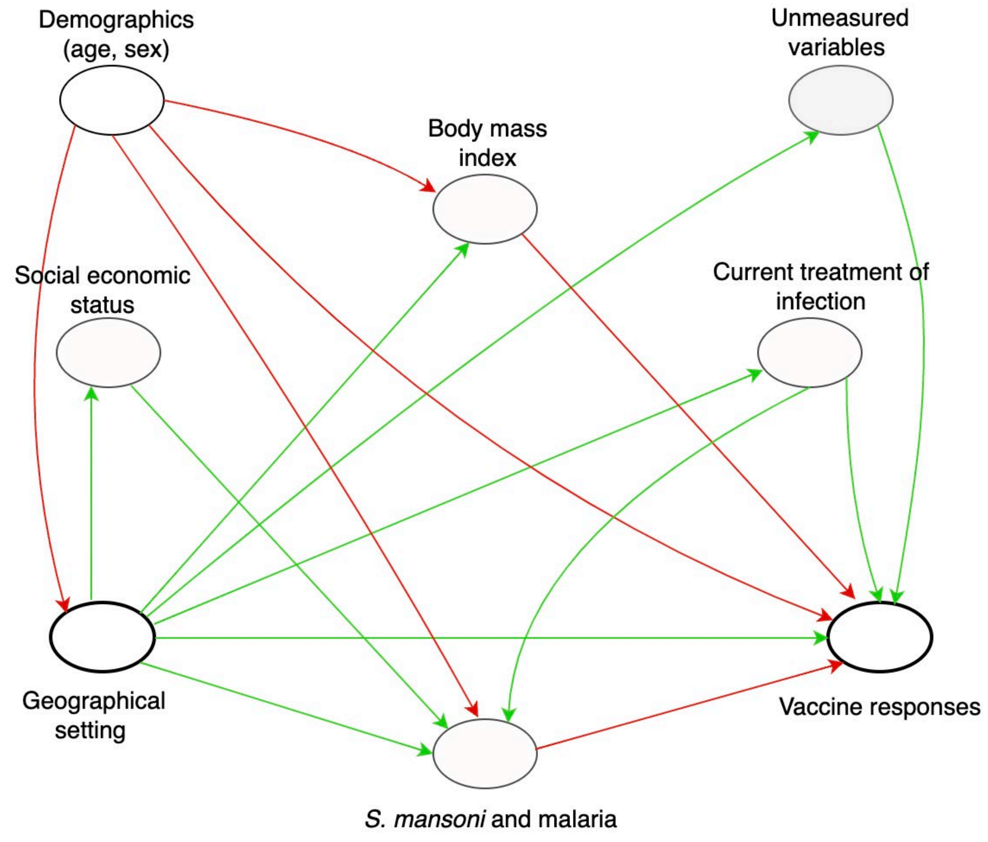

Figure S1: Directed acyclic graph of the hypothesised causal relationships between geographical setting and vaccine responses considering parasitic infections as potential mediators. The direct arrow between geographical setting and vaccine responses depicts the effect though pathways other than the hypothesised mediators. Green arrows show causal paths and red arrows show biasing paths. The minimal sufficient adjustment set for estimating total effects is age and sex. The variables included on the DAG are those where data was collected.

Figure S2: Study profile

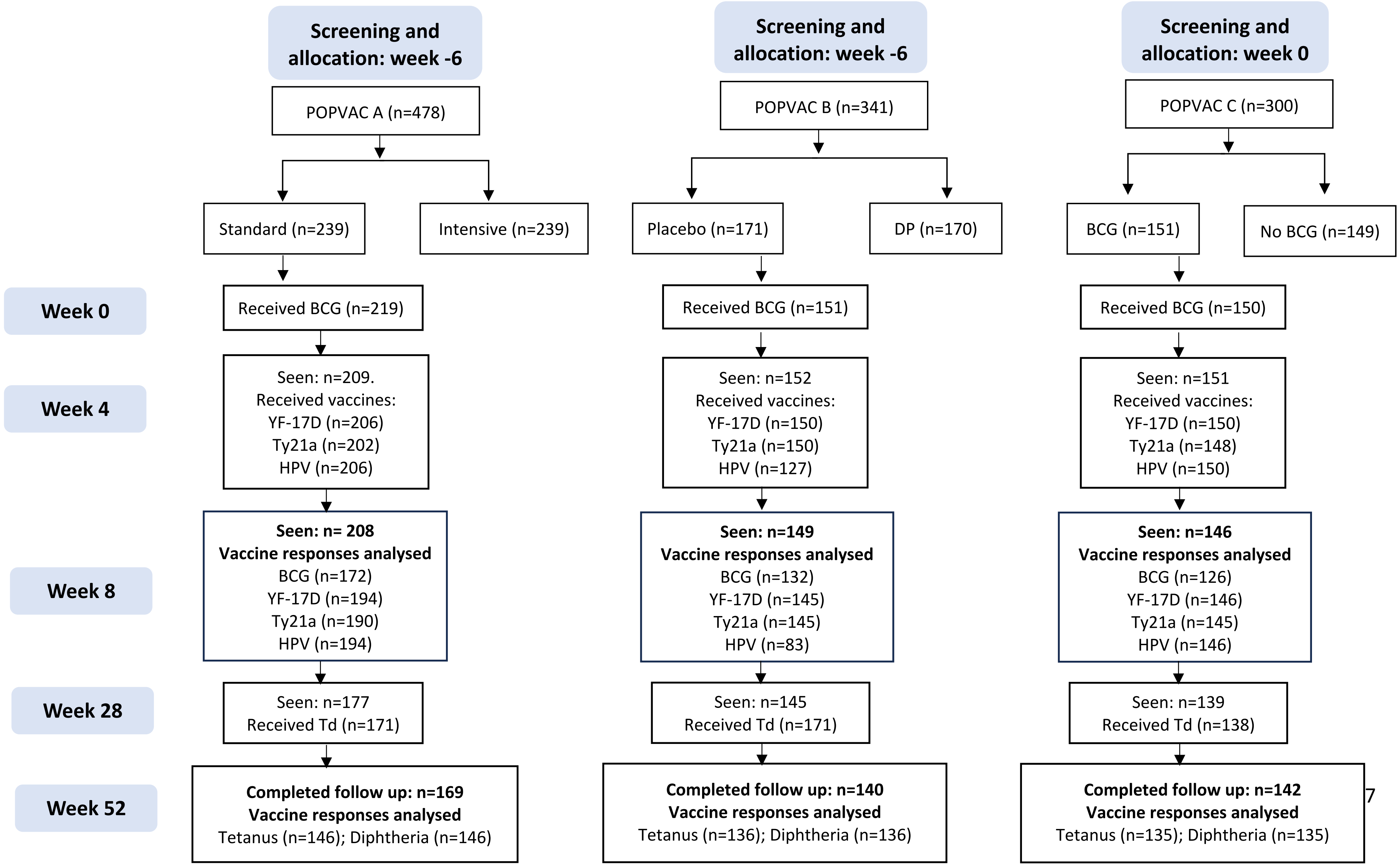

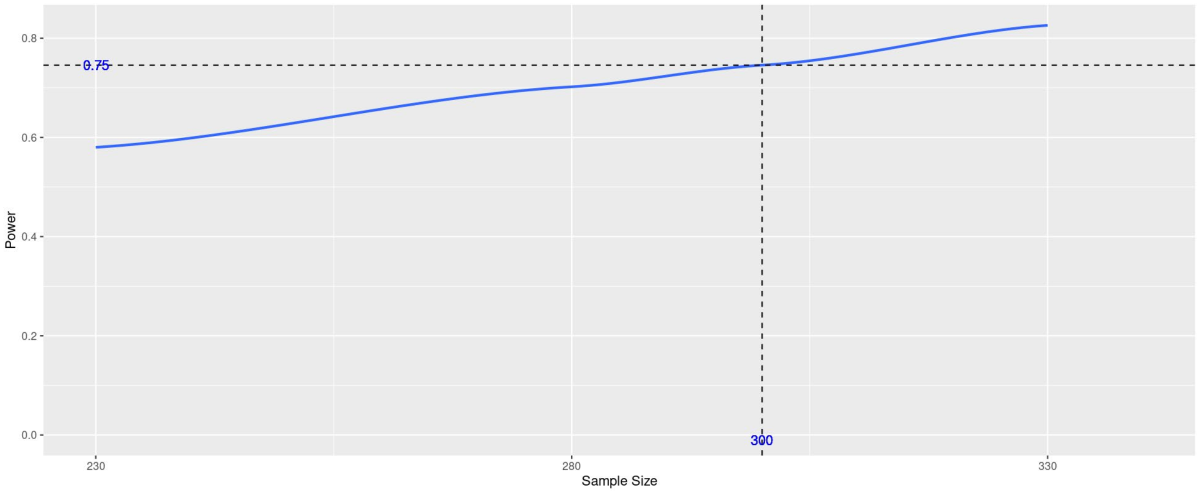

Figure S3: Power estimates for samples sizes ranging from 230 to 330. Sample sizes of 320 and above have at least 80% power to estimate indirect effects lying between 0.038 and 0.068 $\log_{10}$ or bigger (these estimates indicate the average change in the outcome (vaccine response) if the treatment (setting) is held at 0 (set to rural), while the mediator is changed from the level that would be observed when the treatment is 0 to that when the treatment is 1).

Supplementary methods

Inclusion and exclusion criteria

For each trial, participants were included if they attended one of the selected schools or (for POPVAC C) were participants in the EMaBS cohort; they agreed to stay in the study area during the time of the trial, were willing to provide locator information, and for females, agreed to avoid pregnancy during the trial. Exclusion criteria were history of serious psychiatric condition; history of immunodeficiency, endocrine disorder, neurological illness, cancer, or cardiovascular, gastrointestinal, liver, or renal disease; HIV seropositivity, haemoglobin <82 g/L, pregnancy, or current lactation; history of allergic reaction to vaccination or any allergy to any component of the study vaccines; previous YF, oral typhoid or HPV vaccination, or BCG or tetanus and diphtheria vaccination at age ≥ 5 years; tendency to develop keloid scars; acute illness characterised by any of the following: fever, impaired consciousness, convulsions, difficulty in breathing, vomiting; concurrent oral or systemic steroid medication or the concurrent use of other immunosuppressive agents within two months prior to enrolment; use of an investigational medicinal product or non-registered drug, live vaccine, or medical device other than the study vaccines within 30 days prior to dosing with the first study vaccine, or planned use during the study period; administration of immunoglobulins and/or any blood products within the three months preceding the planned trial vaccination date.

Decomposition of the total effect into natural direct and indirect effects

To illustrate the decomposition of NDE and NIE, we define Y as the outcome of interest, A as the exposure, M the mediator and C a set of covariates for each individual. Let Y_a be the counterfactual (potential) outcome for an individual when intervening to set exposure A to a, let M_a denote the counterfactual outcome for M when intervening to set exposure A to a. Let Y_{am} be the counterfactual outcome for Y when exposure A is set to a and mediator M is set to m.

The natural direct effect comparing exposure level A=1 (urban) to A=0 (rural) intervening to fix $M=M_0$ (mediator that would naturally occur for each individual) would be given by

$NDE=Y_1M_0-Y_0M_0$

The natural indirect effect comparing the effects of $M=M_1$ versus $M=M_0$ intervening to fix A=1 would be given by

$NDE=Y_1M_1-Y_1M_0$

The total effect is the sum of the NDE and NIE

$Y_1-Y_0 = Y_1M_1-Y_0M_0 = (Y_1M_1-Y_1M_0) + (Y_1M_0-Y_0M_0)$

Linear regression was used for the outcome model and logistic regression for the mediator models where the mediators were binary and linear regression where mediators were continuous variables.

Since our outcomes were continuous variables, we then defined the regression model for the outcome Y allowing for an interaction between the exposure and mediator as

$E[Y|A=a,M=m,C=c]=\theta_0 + \theta_1 a + \theta_2 m + \theta_3 am + \theta_4' c$

For the binary mediator, the mediator model was specified as $logit[P(M=1|A=a,C=c)]=\beta_0 + \beta_1 a + \beta_2' c$

For the continuous mediator, the mediator model was specified as $E[M|A=a,C=c)]=\beta_0 + \beta_1 a + \beta_2' c$

Plasma detection of *Schistosoma* circulating anodic antigen

Plasma samples collected at screening, week 0, week 8, week 28 and week 52 were retrospectively analysed for *Schistosoma* circulating anodic antigen (CAA) using the up-converting phosphor lateral flow (UCP-LF) assay with a SCAA20 test format with a30 pg/ml positivity threshold[1]. This limit of detection was previously determined by spiking CAA (standard series) in negative serum from non-endemic healthy individuals and analyzed against a large set of confirmed CAA negative controls from different endemic regions. Based on this, quality control was conducted on test materials provided for the study to guarantee a lower limit of detection of 30 pg/ml. All samples above this level (≥30 pg/ml) are regarded as CAA-positive.

For the POPVAC studies, human negative serum (obtained from the Uganda blood bank, Nakasero) was spiked with a known concentration of CAA standard (100,000 pg/ml) and diluted up to eight standard points, with two negative controls. These were used to generate a standard curve to quantify the sample CAA levels: there is no difference between standard curves with spiked CAA in serum and plasma.[1] Plasma and standards (50 µl) were extracted with an equal volume of 4% w/v trichloroacetic acid (TCA; Merck Life Science NV, the Netherlands), vortexed and incubated at ambient temperature for five minutes. Thereafter, samples and standards were briefly vortexed and spun at 13,000 g for five minutes. The resulting supernatant (20µl) was added to the wells containing 100 ng dry UCP particles[2] (400 nm $Y_2O_2S:Yb^{3+}, Er^{3+}$) coated with mouse monoclonal anti-CAA antibodies[3] hydrated with 100µl of high salt lateral flow buffer (HSLF: 200 mM Tris pH8, 270 mM NaCl, 0.5% (v/v) Tween-20, 1% (w/v) BSA). These were incubated for one hour at 37°C while shaking at 900 rpm. The CAA lateral flow strips[4] were labeled with the standard and sample identifications and then placed in the wells on the UCP plate. The samples and standards were allowed to flow and left to dry overnight. The strips were then analysed using the Labrox Upcon reader (Labrox Oy, Turku, Finland). The test line signals (T; relative fluorescent units, peak area) were normalized to the flow control signals (FC) of the individual strips and the results were expressed as ratio values.

Stool PCR detection of Schistosoma mansoni, Necator americanus and Strongyloides stercoralis DNA

Stool samples stored at -80^{0}C in 95% molecular grade ethanol were retrieved and thawed at room temperature. Total DNA (and hence helminth DNA, if present) was extracted from the stool samples using the Fast DNA Spin Kit for Feces (catalogue number 116570200 , MP Biomedicals Germany GmbH) to determine (using multiplex real-time PCR) *S. mansoni, Strongyloides stercoralis* and hookworm (*Necator americanus*) infections.

The DNA extraction procedure was conducted with minor changes to the manufacturer's instructions as follows: samples were left to thaw at room temperature (RT) and then vortexed for five seconds to homogenise the ethanol-stool mixture. The homogenized mixture (0.5 ml) was transferred into a safe-lock microcentrifuge (Eppendorf®) tube and centrifuged at 13000 rpm for 3 minutes to get rid of the ethanol. The pellet was re-suspended in a 2ml lysing matrix E tube, containing 825 µl of sodium phosphate buffer and 275 µl of PLS buffer. For solid samples, 300-500mg of stool were transferred to the lysis tube using a sterile loop. The samples were centrifuged for 5 minutes at 14000 g and the supernatant was decanted. 978 µl Sodium Phosphate Buffer and 122 µL MT Buffer were added to the lysing matrix E tube and vortexed briefly to mix the contents. The samples were then homogenised in the FastPrep® 24 instrument (MP Biomedicals Germany GmbH) at setting 6.0 m/s for 40 seconds and thereafter centrifuged at 14000 g for 5 minutes. The supernatant was then transferred to a clean 2.0 ml centrifuge tube and 250 µl of PPS solution was added. The samples were then shaken vigorously to mix, incubated at 4°C for 10 minutes, and centrifuged at 14000g for 2 minutes. During the centrifugation step, we added 1 ml of binding matrix solution to a clean 15 ml conical tube. The supernatant was then transferred to the 15 ml tube containing the binding matrix. These were mixed gently by hand for 3 to 5 minutes. The samples were then centrifuged at 14000 g for 2 minutes and the supernatant discarded. The binding mixture pellet was then washed by gently re-suspending it in 1 mL Wash Buffer #1. Two spins were performed by first transferring 600 µL of the binding mixture to a filter tube and centrifuged at 14000 g for 1 minute. The catch tube was then emptied and the remaining binding mixture was added to the filter tube and centrifuged as before. The catch tube was emptied again and 500 µL of prepared Wash Buffer #2 (concentrated salt wash solution, reconstituted with absolute molecular grade ethanol) was added to the filter tube and re-suspended by gently pipetting up and down to dislodge the pellet. The samples were centrifuged at the same speed for 2 minutes and the flow-through discarded. The samples were centrifuged again for 2 minutes to extract residual ethanol from the binding matrix and dry the sample. The filter bucket was transferred to a clean 1.9 mL Catch Tube and 100 µL TES elution buffer added. The tube was stirred with a pipette tip to

resuspend the pellet. The samples were then centrifuged for 2 minutes to elute purified DNA into the clean catch tube. The samples were stored at -20°C until used for PCR (below).

The multiplex real-time PCR was adapted from existing procedures.[5,6] Below are the specific forward (F) and reverse (R) primers and TaqMan® probes that were used to simultaneously detect DNA from three helminth species:

Necator americanus

Na58F: 5'-CTGTTTGTCGAACGGTACTTGC-3'

Na158R: 5'- ATAACAGCGTGCACATGTTGC-3'

Nec-2-FAM (MGB): FAM-5'-CTGTACTACGCATTGTATAC-3'-XS

Nec-3-FAM (MGB): 5'-CTGTACTACGCATTGTATGT-3'

Schistosoma mansoni

Ssp48F: 5'-GGTCTAGATGACTTGATYGAGATGCT-3'

Ssp124R: 5'-TCCCGAGCGYGTATAATGTCATTA-3'

Ssp78T-RT: Texas Red-5'-TGGGTTGTGCTCGAGTCGTGGC-3'-BHQ2

Strongyloides stercoralis

Stro18S-1530F: 5'-GAATTCCAAGTAAACGTAAGTCATTAGC-3'

Stro18S-1630R: 5'-TGCCTCTGGATATTGCTCAGTTC-3'

Stro-4-TRBhq2-VIC- 5'-ACACACCGSCCGTCGCTGC-3'

Phocine herpes virus (PhHV) DNA, extracted from the Phocine herpes virus (kindly provided by Dr. Martin Schutten, Erasmus Medical Center, Rotterdam, the Netherlands), was included in the PCR master mix, thus distributed to all reaction wells as an internal control to check for PCR inhibition. The PhHV forward primer PhHV-267s (5'-GGGCGAATCACAGATTGAATC-3'), reverse primer PhHV-337as (5'-GCGGTTCCAAACGTACCAA-3') and probe PhHV-305tq (Cy5-5' TTTTTATGTGTCCGCCACCATCTGGATC-3'-BHQ2) were used for Phocin herpes virus DNA detection. A positive pool was included on the plate for every run as a test control. The positive pool was made up of a mixture of DNA from samples (from among the study samples) that were highly positive for *S. mansoni* and *N. americanus* on Kato-Katz, and for *S. stercoralis* by PCR (conducted on samples from a previous study). The amplification conditions were 15 minutes at 95°C, 50 cycles of 15 seconds at 95°C, 30s at 60°C and 30s at 72°C. DNA amplification, detection and data analysis were attained with the ABI 7500 Fast Real time machine and 7500 Fast systems software version 1.5.1.

***Ex vivo* interferon-γ ELISpot assays to quantify BCG-specific responses**

To quantify BCG-specific responses, we conducted *ex vivo* interferon (IFN)-γ ELISpot assays, using a Human IFN-γ (ALP) ELISpot Flex kit (Mabtech, Sweden) and multiscreen-IP 0.45μm filter 96-well plates (Merck Millipore). ELISpot plates were coated overnight at 4°C with 50 μl of anti-IFN-γ capture antibody (15 μg/ml) dissolved in 0.05 M carbonate-bicarbonate buffer (Sigma Aldrich). The plates were then washed 5 times with sterile 1X PBS (Sigma Aldrich), and blocked (2-5 hours, 37°C) by adding 100 μl/well of R10 medium (10% fetal bovine serum [Sigma Adrich] in RPMI 1640 medium [Thermofisher scientific] supplemented with L-glutamine, streptomycin, HEPES buffer and penicillin [all from Life technologies, UK]). During plate blocking, peripheral blood mononuclear cells (PBMCs) were isolated from heparinised whole blood by density gradient centrifugation with Histopaque® (Sigma Aldrich). For each study sample, PBMCs (300,000 per test well) were stimulated in duplicate for 18-20 hours at 37°C, 5% CO_2, with BCG (Moscow strain, Serum Institute of India) at a concentration of 200,000 colony forming units per ml, or left unstimulated. Staphylococcal enterotoxin B (SEB; Sigma Aldrich) was used at a final concentration of 10 μg/ml as a positive control, and a 1:1 mix of the 6-kDa early secretory antigenic target and 10-kDa culture filtrate protein (ESAT-6 and CFP-10 recombinant proteins from BEI Resources, USA) used at a final concentration of 2.5 μg/ml for exploratory assessment of tuberculosis infection. Following the 18-20 hour incubation, plates were washed 5 times with 200ul/well PBS containing 0.05% Tween 20 (Sigma Aldrich) and incubated for 2 hours at room temperature with 50 μl per well of a 1/1000 PBS dilution of biotin anti-IFN-γ antibody from the ELISpot kit. After another washing step with PBS-0.05% Tween 20, plates were incubated for 1 hour at room temperature with 50 μl per well of a 1/1000 PBS dilution of a streptavidin-alkaline phosphatase conjugate from the ELISpot kit. Plates were washed, developed for 3-10 minutes with 50 μl per well of 5-Bromo-4-chloro-3-indoxyl phosphate/Nitro blue tetrazolium (BCIP/NBT; Europa Bioproducts), and the reaction stopped by washing the plate under tap water. Plates were allowed to dry in the dark at room temperature and read using an ELISpot reader (Autoimmun Diagnostika Gmbh iSpot , Strassberg, Germany) running AID ELISpot software v.7.0. Spot-forming units (SFUs) per well were manually verified to remove artefacts.

We performed QC through a number of steps: 1) for each sample, we checked to ascertain whether the PBMC isolation procedure was conducted within eight hours after sample collection; 2) we inspected each ELISpot plate visually for quality and completeness of labelling (sample IDs, date, time point and antigens) and compared the plate picture to the exported spot count spreadsheet to ensure the correct data had been exported; 3) we checked the calculated data in the exported database to ensure the background subtraction, average of duplicate wells and multiplication up to

spot forming units per million PBMC had been performed correctly; 4) we assessed whether the unstimulated well and SEB well controls for each assay were within the accepted range.

Results were reported as SFUs per a million PBMCs, calculated sequentially by 1) subtracting mean SFUs of unstimulated wells from mean SFUs of duplicate antigen wells, and 2) correcting for the number of PBMCs per well (300,000). Samples that had more than 83.3 SFUs per a million PBMCs in the unstimulated well were considered invalid and not included in the final analysis.

Yellow Fever plaque reduction neutralizing reference test (PRNT)

A plaque reduction assay as described by Beaty *et al*.[7] was used. Briefly, Vero cells at a concentration of 65,000 cells/ml were seeded into 6-well plates (Greiner Bio-One GmbH, Germany) at a volume of 3 ml/well. Cells were cultured in growth medium (1X Eagle's Minimum Essential Medium, 8% heat inactivated fetal bovine serum, 100 units penicillin/streptomycin, gentamycin 50 mg/ml and fungizone 1 mg/ml) at 37°C (with 5% CO_2) for 3–4 days. Culture medium was then removed from the cell monolayer by dumping. Test plasma were inactivated at 56°C for 30 min to remove complement factor, serially (two-fold) diluted from 1:10 to 1:20480 in BA-1 diluent (10X M199 Hanks' Salts without L-Glutamine, 5% Bovine Serum Albumin, 1M TRIS-HCL pH 7.5, L-Glutamine, sodium bicarbonate 7.5%, 100X penicillin/streptomycin, 1000X fungizone in sterile water), and mixed with approximately 200 Plaque Forming Units (PFU) of a reference YF-17D virus preparation. The plasma-virus mixture (0.1 ml) was added to the confluent monolayer of Vero cells in each well and incubated at 37°C (with 5% CO_2) for 1 hour. The first overlay medium (comprising Miller's 2X Yeast Extract-Lactalbumin hydrolysate medium, 10X Earle's Buffered Salts Solutions, 2% fetal bovine serum, 1000X fungizone, 1000X Gentamycin, and 2% low-melting agarose) was added, 3 ml per well, and allowed to solidify for 30 minutes at room temperature. The plates were incubated at 37°C with 5% CO_2 for 4 days. To stain cell layers, a Neutral Red dye (Sigma Aldrich) second overlay was added, 2 ml per well, and allowed to solidify for 30 minutes at room temperature. After this second overlay, plates were incubated at 37°C in 5% CO_2 for 2 days: plaques were counted first on day 1 and the final score documented on day 2 to establish 50% and 90% neutralization titres. Back titration plates were established to ensure infectivity of cell monolayer and standardization of virus to 200 PFU/0.1 ml. Neutralisation titres <1:10 were considered negative. Titres of 1:10 were interpreted as borderline. The PRNT antibody titres presented refer to the reciprocal of the last plasma dilution that reduced by 50% (PRNT50) or 90% (PRNT90) the number of virus plaque clusters infected by 100 PFU/0.1 ml of the reference 17D virus preparation.

For quality control, we used a high titre positive control (PC), with the last six titre dilution range, from 640 to 20480. So long as the PC titre was within the expected range and did not vary by greater than 4-fold, the assay passed quality control. Furthermore, we ran back-titrations of the virus

inoculum (standardised to 200 PFU/0.1 ml) to determine the end-point specimen antibody titre at 50% or 90% neutralisation. The number of virus plaques infected at 50% neutralisation and at 90% neutralisation were expected to be within an approximate range of 25-100 and 5-20, respectively.

Detection of plasma IgG against *Salmonella* Typhi O-lipopolysaccharide (O:LPS)

Specific IgG to *S.* Typhi O-lipopolysaccharide (O:LPS) was measured by an in-house ELISA. Nunc Maxisorp 96-well plates (Thermo Fisher) were coated overnight at 4ºC with 50µl/well of 10 µg/ml of *S.* Typhi O:LPS (Sigma L2387) in bicarbonate (Na_2CO_3 + $NaHCO_3$) buffer (0.1M, pH 9.6). Plates were washed with phosphate-buffered saline (PBS 1X)-Tween 20 (0.05%) solution, blocked with 200µl of 5% skimmed milk diluted in PBS-Tween 20 for 1 hour at room temperature (RT), washed again and incubated for 2 hours at RT with 50µl of test plasma samples (diluted 1/320 with 1% skimmed milk in PBS-Tween 20) and two-fold serially diluted standard sera (top concentration 20 EU/ml). Standards were derived from a pooled sample generated from sera of known O:LPS-specific IgG titres, kindly provided by the Oxford Vaccine Centre Biobank. These sera had been collected from the highest responders to O-antigen following challenge with *S.* Typhi in a controlled human infection study.[8] Following test and standard sample incubation, plates were washed and O:LPS-specific IgG binding detected by incubating the plates for 1 hour at RT with goat anti-human IgG-horseradish peroxidase conjugate (Insight Biotechnology, UK), diluted 1/6000 in 1% skimmed milk–PBS-Tween 20. Plates were washed and developed by addition of 100µl of o-phenylenediamine (Sigma-Aldrich) and reactions stopped after 5 minutes with 30µl of 2M Sulphuric acid. Optical density (OD) values were measured at 490nm (reference wavelength 630nm) on a 96-well plate ELISA reader (BioTek ELx808, USA). Nominal ELISA units (EU/ml), representing O:LPS-specific IgG titres, were interpolated from standard curves using a five-parameter curve fit using Gen5 data collection and analysis software (BioTek Instruments Inc, Vermont, Winooski, USA).

Detection of plasma IgG against Human Papillomavirus type 16 (HPV-16) and HPV-18

Anti-HPV-16 and HPV-18 IgG concentrations were measured by ELISA, as previously described.[9-12] Nunc Maxisorp 96-well plates (Thermo Fisher) were coated with 100 µl of HPV-16 L1 virus-like particles (VLP) at a concentration of 2.7 µg/ml, or with HPV-18 L1-L2 VLP at a concentration of 2 µg/ml and incubated at 4°C overnight. Plates were washed three times with a 1X phosphate-buffered saline (PBS)-Tween 20 (0.25%) solution, and blocked for 90 minutes at room temperature (RT) with 4% skimmed milk diluted in a 1X PBS-0.25% Tween 20 solution. The plates were washed three times and and incubated (with gentle shaking) for 1 hour at RT with 100 µl of test plasma samples, assay controls and two-fold serially diluted standard sera. Pre-vaccination test plasma samples were diluted 1/50 (HPV-16 assay) or 1/200 (HPV-18 assay) with blocking buffer, while post-vaccination plasma samples were diluted 1/400 for both HPV-16 and HPV-18 assays. Standard sera

were used at a top concentration of 1.28 EU/ml and 8.2425 EU/ml for HPV-16 and HPV-18 assays, respectively. Following test and standard sample incubation, plates were washed four times and further incubated for 1 hour at RT with peroxidase-labeled goat anti-human IgG (KPL, Gaithersburg, Maryland). Plates were then developed with a tetramethylbenzidine substrate solution (KPL, Inc.) for 25 minutes in the dark at room temperature. Next, the reaction was stopped by adding 100 µl of 0.36N H_2SO_4 to each well. Optical density (OD) values were measured at 450 nm (reference wavelength 630 nm) on a 96-well plate ELISA reader (BioTek ELx808, USA). Nominal ELISA units (EU/ml), representing HPV-16 L1 VLP- and HPV-18 L1-L2 VLP-specific IgG titres, were interpolated from standard curves using a five-parameter curve fit using Gen5 data collection and analysis software (BioTek Instruments Inc, Vermont, Winooski, USA).

For quality control (QC), the acceptable R^2 for the standard curve was ≥ 0.990 and the average optical density (OD) range of the top standard was 2.0-4.0. The acceptable OD of the last (8th) standard dilution was ≤0.25. The percentage difference in ODs between standard dilutions (i.e. n and n+1 dilution) was expected to be ≥ 0.3. Plates were repeated if they failed to meet these standard curve criteria. The calculated negative control cut-off was 4±3 EU/ml and 60±10 EU/ml for HPV-16 and HPV-18, respectively. Plates whose negative control concentration was above the cut-offs were repeated. The calculated positive control concentration was 450±10 EU/ml and 4500±10 EU/ml for HPV-16 and HPV-18 respectively. Background signal was measured by a blank whose OD was expected to be ≤0.05. Higher ODs indicated assay contamination and plates were repeated.

ELISA measurement of anti-diphtheria and anti-tetanus IgG

Nunc Maxisorp 96-well plates (Thermo Fisher) were coated with 50 µl of either 2 Lf units per ml of diphtheria toxoid (NIBSC product code 13/212) per ml or 0.56 Lf units per ml of tetanus toxoid (NIBSC product code 02/232) in Na_2CO_3/$NaHCO_3$ buffer (0.1M, pH 9.6) overnight at 4°C. Plates were washed with 0.05% Tween 20 in 1X phosphate-buffered saline (PBST) and blocked for 1 hour with 5% skimmed milk powder in PBST at 37 °C. The plates were washed four times and and incubated for 2 hours at 37 °C with 50 µl of test plasma samples, and serially diluted WHO International Standard anti-toxins for diphtheria (NIBSC 10/262) or tetanus (NIBSC 13/240). Samples and standards were prepared in PBST + 1% skimmed milk (assay buffer). Pre-vaccination test samples were added at a dilution of 1/150, while post-vaccination plasma samples were added at a dilution of 1/300 in assay buffer. The standards were used at a top concentration of 3 IU/ml and 0.125 IU/ml for the tetanus and diphtheria assays, respectively. Plates were washed four times and incubated for 1 hour at 37 °C with 50 µl of polyclonal rabbit anti-human IgG HRP-conjugate (Agilent Dako, CA, USA) diluted 1/3000 in assay buffer. After another washing step, plates developed by adding 100 µl/well of o-phenylenediamine (Sigma-Aldrich) and reactions stopped after 5 minutes with 25 µl/well of 2M

sulphuric acid. Optical density (OD) values were measured at 490nm (reference wavelength 630nm) on a 96-well plate ELISA reader (BioTek ELx808, USA). Tetanus and diphtheria toxoid-specific IgG concentrations (IU/ml) were interpolated from standard curves using a five-parameter curve fit using Gen5 data collection and analysis software (BioTek Instruments Inc, Vermont, Winooski, USA).

Detection of *Plasmodium falciparum* DNA by PCR

Prior to the PCR, DNA extraction was performed using the QIAamp DNA Blood Mini Kit (Catalogue number 51106, QIAGEN). Briefly, whole blood pellet samples stored at -80^{0}C were retrieved and thawed at room temperature. Reagents in the extraction kit were reconstituted as per the manufacturer's instructions. Volumes of retrieved samples were checked and Phosphate Buffered Saline (PBS) was added to samples with inadequate volumes. These were vortexed thoroughly for 2 to 3 minutes. The protease enzyme (20 µl) was pipetted to the bottom of a 2 ml Eppendorf tube, and the whole blood pellet (200 µl) was transferred to the tube. The AL lysis buffer (200 µl), after thorough mixing, was added and pulse vortexed for 20 seconds. The samples were then placed in a heating block set to 56^{0}C for 15 minutes and thereafter centrifuged at 6000g for 1 minute to ensure no sample was trapped in the Eppendorf tube lid. 200 µl of absolute molecular grade ethanol was added to the sample, pulse vortexed, and then centrifuged. 500 µl of the sample was transferred to a spin column and centrifuged at 6000g for 1 minute, discarding the flow-through thereafter. This process was repeated for the remainder of the sample. The DNA was then purified using consecutive washes with AW1 (500ul, 250ul) and AW2 (500ul) buffers. The QIAGEN AE buffer (150 µl) was used to elute the DNA. The samples were then stored at -20^{0}C until used in the PCR.

The real-time PCR was performed with the ABI 7500 Fast Real-time machine and data processed using 7500 Fast Systems software version 1.5.1. The PCR reaction was performed with a final volume of 25 µl containing 2 µl of DNA 1 µl of PhHV DNA (as an internal control, detailed below) and 22 µl of PCR master mix made of HotStarTaq Master Mix (Catalogue Number 203446), primers, and probes. The primers and probes used in this study are listed below.

PFal-F 5`-CCG ACT AGG TGT TGG ATG AAA GTG TTA A-3`
Plas-171R 5`-AAC CCA AAG ACT TTG ATT TCT CAT AA-3
Pfal114-XS_YY 5`-CTT TCG AGG TGA CTT TTA GAT-3`-BHQ1

Phocine herpes virus (PhHV) DNA, extracted from the Phocine herpes virus (kindly provided by Dr. Martin Schutten, Erasmus Medical Center, Rotterdam, the Netherlands), was included in the PCR master mix, thus distributed to all reaction wells as an internal control to check for PCR inhibition. The PhHV forward primer PhHV-267s (5'-GGGCGAATCACAGATTGAATC-3'), reverse primer PhHV-337as (5'-GCGGTTCCAAACGTACCAA-3') and probe PhHV-305tq (Cy5-5'-

TTTTTATGTGTCCGCCACCATCTGGATC-3'-BHQ2) were used for Phocin herpes virus DNA detection. A pool of DNA extracted from *P. falciparum* positive samples was used to set serially diluted standards tested alongside the samples on every plate run. The amplification conditions were 15 minutes at 95°C, 50 cycles of 15 seconds at 95°C, 30s at 60°C, and 30s at 72°C.

Detection of IgG against *P. falciparum* merozoite surface protein-2 (MSP-2) and *P. falciparum* apical membrane antigen-1 (AMA-1)

Specific IgG to *P. falciparum* merozoite surface protein-2 (MSP-2) and *P. falciparum* apical membrane antigen-1 (AMA-1) was measured in plasma samples by ELISA as previously described.[2,3,14] Immulon4 HBX 96-well plates (Thermo Fisher) were coated overnight at 4°C with 50µl/well of 0.13 µg/ml of *Pf*AMA-1 or 0.23µg/ml *Pf*MSP-2 in bicarbonate (Na_2CO_3 + $NaHCO_3$) buffer (0.1M, pH 9.6). Plates were washed with phosphate-buffered saline (PBS 1X)-Tween 20 (0.05%) solution, blocked with 150µl of 1% skimmed milk diluted in PBS-Tween 20 for 3 hours at room temperature (RT), washed again and incubated for overnight at +4°C with 50µl of test plasma samples (diluted 1/2000 for *Pf*AMA-1 or 1/1000 *Pf*MSP-2 with PBS-Tween 20) and four-fold serially diluted standard sera (pooled positive serum from high transmission area; starting with 1/100 for *Pf*AMA-1 or 1/50 for *Pf*MSP-2). Following test and standard sample incubation, plates were washed, and antigen-specific IgG binding detected by incubating the plates for 3 hours at RT with 50µl rabbit anti-human IgG-horseradish peroxidase conjugate (Dako), diluted 1/5000 in 1% skimmed milk–PBS-Tween 20. Plates were washed and developed by addition of 100µl of o-phenylenediamine (Sigma-Aldrich) for 10-15 minutes and reactions stopped with 25µl of 2M Sulphuric acid. Optical density (OD) values were measured at 490nm using a 96-well plate ELISA reader (BioTek ELx808, USA). Optical densities for *Pf*AMA-1 and *Pf*MSP-2 in test samples and standards were obtained by subtracting mean optical densities of duplicate blank wells from mean optical densities of duplicate test samples and standards. Arbitrary ELISA units, representing *Pf*AMA-1 and *Pf*MSP-2 -specific IgG titres, were interpolated from standard curves for ODs using Microsoft Excel.

Schistosoma egg antigen (SEA)-specific IgG ELISA

All but the first two columns of 4HX Immulon (VWR, UK, Cat No 735-0465) 96-well plates were coated with 50µl of SEA [2.4 µg/ml] (purchased from Professor Michael J Doenhoff, University of Nottingham) in bicarbonate (Na_2CO_3 + $NaHCO_3$) buffer (0.1M, pH 9.6). Two-fold serial dilutions of purified human IgG control (whole molecule, GenScript), diluted in bicarbonate buffer, were added to the first two columns of each plate to form standard curves. The top standard concentration was 4 µg/ml. The plates were then incubated overnight at 4°C. Plates were washed with phosphate-buffered saline (PBS)-Tween 20 solution, blocked with 200µl of 5% skimmed milk diluted in PBS-Tween 20 at room temperature (RT), and incubated for two hours at RT with 50µl of plasma samples

diluted 1/500 with 1% skimmed milk in PBS-Tween 20 (assay buffer). Plates were washed and antibody binding detected by incubating the plates for one hour at RT with a 1/3000 dilution of polyclonal rabbit anti-human IgG/HRP (Dako, Denmark). Plates were developed by addition of 100µl of o-phenylenediamine (Sigma-Aldrich) and reactions stopped after five minutes with 30µl of 2M Sulphuric acid. Optical density values were measured at 490nm (reference wavelength 630nm) on a 96-well plate ELISA reader. IgG concentrations (µg/ml) were interpolated from standard curves using a five-parameter curve fit using Gen5 data collection and analysis software (BioTek Instruments Inc, Vermont, Winooski, USA).

References

1. Corstjens PL, De Dood CJ, Kornelis D, et al. Tools for diagnosis, monitoring and screening of Schistosoma infections utilizing lateral-flow based assays and upconverting phosphor labels. *Parasitology* 2014; **141**(14): 1841-55.
2. Corstjens PL, Li S, Zuiderwijk M, et al. Infrared up-converting phosphors for bioassays. *IEE Proc Nanobiotechnol* 2005; **152**(2): 64-72.
3. Corstjens PL, van Lieshout L, Zuiderwijk M, et al. Up-converting phosphor technology-based lateral flow assay for detection of Schistosoma circulating anodic antigen in serum. *Journal of clinical microbiology* 2008; **46**(1): 171-6.
4. Corstjens P, Hoekstra PT, de Dood CJ, van Dam GJ. Utilizing the ultrasensitive Schistosoma up-converting phosphor lateral flow circulating anodic antigen (UCP-LF CAA) assay for sample pooling-strategies. *Infect Dis Poverty* 2017; **6**(1): 155.
5. Verweij JJ, Brienen EA, Ziem J, Yelifari L, Polderman AM, Van Lieshout L. Simultaneous detection and quantification of Ancylostoma duodenale, Necator americanus, and Oesophagostomum bifurcum in fecal samples using multiplex real-time PCR. *The American journal of tropical medicine and hygiene* 2007; **77**(4): 685-90.
6. Verweij JJ, Canales M, Polman K, et al. Molecular diagnosis of Strongyloides stercoralis in faecal samples using real-time PCR. *Transactions of the Royal Society of Tropical Medicine and Hygiene* 2009; **103**(4): 342-6.
7. Beaty BJ, Calisher CH, Shope RE. Arboviruses. In: Schmidt NJ, Lennette DA, Lennette ET, Lennette EH, Emmons RW, eds. Diagnostic Procedures for Viral, Rickettsial and Chlamydial Infections. 7th ed. Washington DC: American Public Health Association; 1995: 204-5.
8. Gibani MM, Jin C, Shrestha S, et al. Homologous and heterologous re-challenge with Salmonella Typhi and Salmonella Paratyphi A in a randomised controlled human infection model. *PLoS neglected tropical diseases* 2020; **14**(10): e0008783.

9. Dauner JG, Pan Y, Hildesheim A, Kemp TJ, Porras C, Pinto LA. Development and application of a GuHCl-modified ELISA to measure the avidity of anti-HPV L1 VLP antibodies in vaccinated individuals. *Mol Cell Probes* 2012; **26**(2): 73-80.

10. Dauner JG, Pan Y, Hildesheim A, Harro C, Pinto LA. Characterization of the HPV-specific memory B cell and systemic antibody responses in women receiving an unadjuvanted HPV16 L1 VLP vaccine. *Vaccine* 2010; **28**(33): 5407-13.

11. Pinto LA, Kemp TJ, Torres BN, et al. Quadrivalent Human Papillomavirus (HPV) Vaccine Induces HPV-Specific Antibodies in the Oral Cavity: Results From the Mid-Adult Male Vaccine Trial. *The Journal of infectious diseases* 2016; **214**(8): 1276-83.

12. Miller CN, Kemp TJ, Abrahamsen M, et al. Increases in HPV-16/18 antibody avidity and HPV-specific memory B-cell response in mid-adult aged men post-dose three of the quadrivalent HPV vaccine. *Vaccine* 2021; **39**(37): 5295-301.

13. Bousema T, Youssef RM, Cook J, et al. Serologic markers for detecting malaria in areas of low endemicity, Somalia, 2008. *Emerg Infect Dis* 2010; **16**(3): 392-9.

14. Ndungu FM, Mwacharo J, Wambua J, et al. A seven-year study on the effect of the pre-erythrocytic malaria vaccine candidate RTS,S/AS01 (E) on blood stage immunity in young Kenyan children. *Wellcome Open Res* 2019; **4**: 42.
